# Harnessing Piezoelectric Biomaterials for Pathogenic Eradication and Tissue Regeneration

**DOI:** 10.1002/EXP.20240315

**Published:** 2026-01-28

**Authors:** Wenxuan Mao, Xiaoye Li, Ao He, Meng Ding, Yu Zhang, Zhuo Dai, Qiang Li, Weijun Xiu, Yanling Hu, Yongbin Mou, Dongliang Yang, Heng Dong

**Affiliations:** ^1^ Institute of Stomatology Nanjing Stomatological Hospital Affiliated Hospital of Medical School Nanjing University Nanjing China; ^2^ Institute For Health Innovation and Technology Biomedical Engineering Department National University of Singapore Singapore; ^3^ Nanjing Polytechnic Institute Nanjing China; ^4^ Key Laboratory of Flexible Electronics (KLOFE) and Institute of Advanced Materials (IAM) School of Physical and Mathematical Sciences Nanjing Tech University (NanjingTech) Nanjing China

**Keywords:** antibacterial, piezoelectric biomaterials, piezoelectric effect, tissue regeneration

## Abstract

The increasing incidence of infections and impaired tissue healing underscores the urgent need for effective therapeutic strategies for antibacterial treatment and regenerative medicine. Piezoelectric catalytic therapy represents an innovative approach that converts mechanical energy into electrochemical energy, providing a versatile platform for biomedical applications. However, conventional piezoelectric materials face significant limitations, including poor biocompatibility and insufficient catalytic efficiency, restricting their clinical translation. Piezoelectric biomaterials, characterized by excellent biocompatibility and enhanced piezoelectric performance, have recently emerged as promising candidates to overcome these challenges. This review systematically summarizes the latest advancements in piezoelectric biomaterials designed for pathogenic eradication and tissue regeneration. We provide a comprehensive overview of piezoelectric biomaterial classifications, fundamental mechanisms of piezoelectric catalysis, and methods to enhance material properties. Furthermore, we explore current applications of piezoelectric biomaterials in antibacterial therapies and regenerative medicine. Finally, critical challenges and potential future directions in material optimization and clinical application are identified, aiming to stimulate further innovation and research in this transformative field.

## Graphical abstract

This review highlights recent advances in piezoelectric biomaterials for antibacterial therapy and tissue regeneration, discussing material classifications, catalytic mechanisms, property enhancements, biomedical applications, and future challenges in clinical translation.

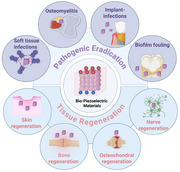

## 1 Introduction

Pathogenic infections remain a persistent global health challenge, ranging widely from mild skin irritation to severe conditions, leading to substantial morbidity, mortality, and economic burdens worldwide. Traditional antibacterial materials, such as antibiotics and metal‐based agents, have been instrumental in combating bacterial infections [1]. However, their efficacy is increasingly compromised by several limitations. The overuse and misuse of antibiotics have led to the emergence of multidrug‐resistant bacteria, commonly known as “superbugs,” rendering many conventional treatments ineffective. Additionally, metal‐based antibacterial agents, while effective, often pose cytotoxicity risks and environmental concerns because of the release of metal ions, such as sliver (Ag) ion. Furthermore, these traditional materials may lack specificity, leading to undesirable effects on beneficial microbiota. In contrast, piezoelectric materials have emerged as promising alternatives in antibacterial applications. These materials generate internal electric fields in response to mechanical stimuli, such as ultrasound (US) or physiological movements, promoting electron–hole pair separation and subsequent generation of reactive oxygen species (ROS) that can effectively disrupt bacterial cells [2, 3]. Unlike conventional antibiotics, piezoelectric catalysts mechanically disrupt bacterial membranes and biofilms, thus reducing toxicity and resistance [4]. In addition, the antibacterial activity of piezoelectric materials can be precisely controlled through external mechanical stimuli, allowing for localized treatment and minimizing damage to surrounding healthy tissues. These attributes position piezoelectric materials as innovative and effective solutions for addressing the challenges posed by resistant bacterial infections.

In addition to combating infections, piezoelectric materials offer substantial promise for tissue regeneration therapies, which are increasingly critical owing to the rising incidence of tissue damage resulting from injuries and diseases. Current therapeutic strategies frequently fail to achieve complete and efficient healing and functional restoration, underscoring the urgent need for innovative technologies that actively support cellular activities and tissue repair. Piezoelectric materials uniquely influence the cellular behaviors essential for regeneration, including cell migration, proliferation, and differentiation. Through the piezoelectric effect, these materials generate interfacial electrical charges that interact directly with biological processes, modulating molecular sensors such as ion channels, cytoskeletal components, cell adhesion proteins, and various signaling pathways. This modulation promotes cell activities and accelerates regeneration in bone, nerve, and tendon tissues, significantly enhancing tissue repair [5]. Moreover, piezoelectric materials not only regulate cellular behavior but also serve as scaffolds that facilitate cell adhesion and provide structural support, further enhancing their application potential in tissue engineering [6].

Owing to the burgeoning application potential of piezoelectric materials in biomedicine, an increasing number of researchers are dedicated to the development of piezoelectric biomaterials that exhibit superior biocompatibility. Traditional piezoelectric materials, such as lead zirconate titanate (PZT), are recognized for their outstanding piezoelectric properties [7]. However, their high lead content presents significant concerns regarding their biocompatibility and safety. As a result, substantial research has shifted towards lead‐free piezoelectric materials, including barium titanate (BaTiO_3_, BTO), zinc oxide (ZnO), and bismuth ferrite (BiFeO_3_). Modification strategies, such as surface engineering, defect incorporation, polymer composite, and heterojunction design, have effectively enhanced their piezoelectric performance, making them comparable to that of traditional materials. These lead‐free alternatives not only provide enhanced environmental sustainability but also offer improved biocompatibility, thereby advancing forward the development of piezoelectric biomaterials specifically engineered for biomedical applications.

**FIGURE 1:**
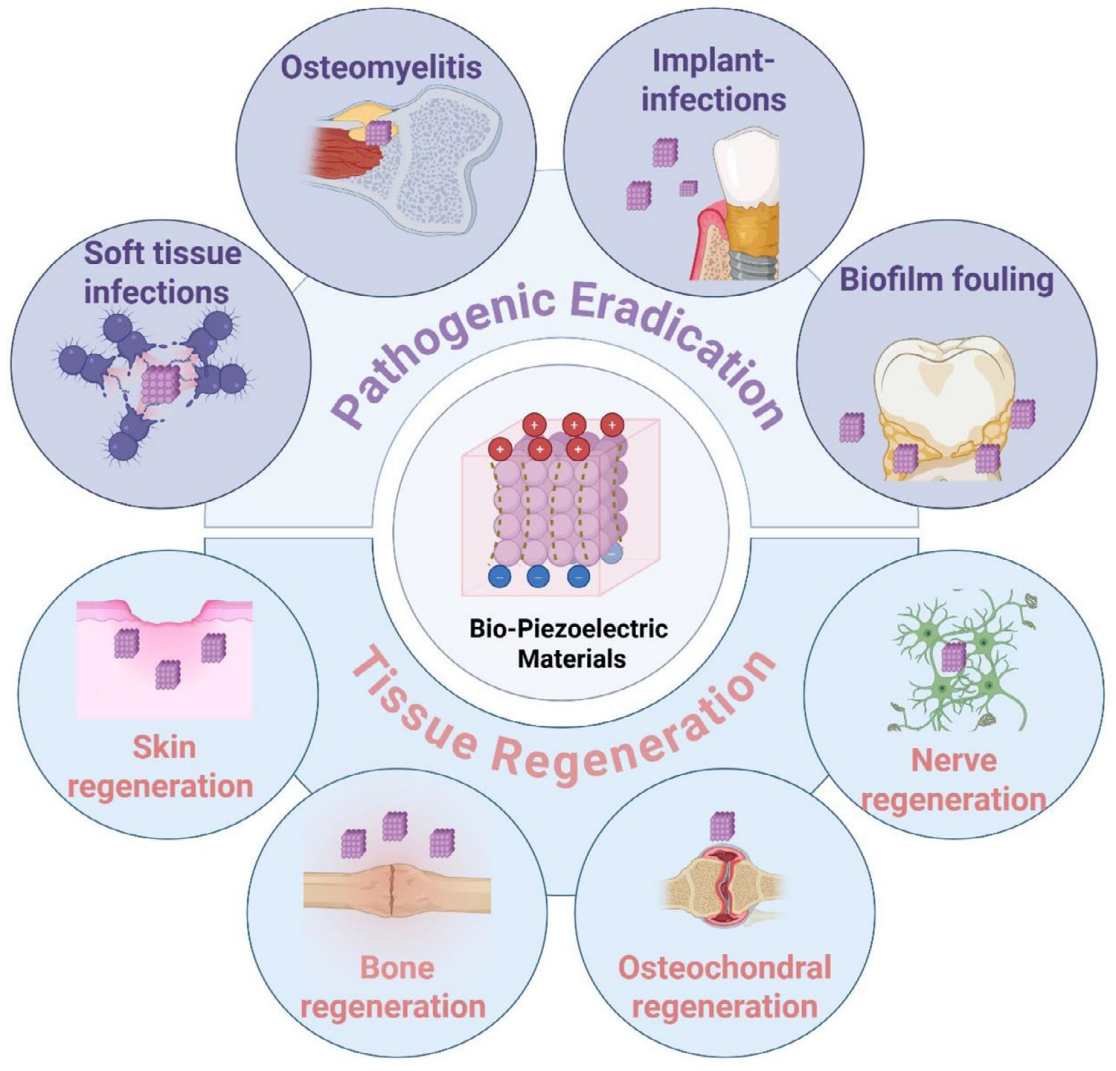
Schematic of the biomedical applications of piezoelectric biomaterials.

Herein, this review offers a detailed conclusion of the use of piezoelectric biomaterials for catalysis in the medical field. It commences by classifying piezoelectric biomaterials based on their structures, material origins and morphological forms. Subsequently, it subsequently delves into the principles of piezoelectric catalysis, along with various strategies for enhancing piezoelectric properties. Next, this review focuses on the application of piezoelectric biomaterials in pathogen eradication and tissue regeneration (Figure 1). For pathogen eradication, the focus is on their antibacterial capabilities (targeting free bacteria and biofilms) and their effectiveness in treating infectious diseases such as osteomyelitis and implant infections. With respect to tissue regeneration, this review explores their roles in the regeneration of skin, bone, cartilage, nerve, and other tissues. Finally, it concludes by discussing the ongoing challenges and future directions in the development of piezoelectric biomaterials and their applications in catalysis and regenerative medicine. This review aims to offer a unique and thorough perspective to inspire further research in antibacterial and regeneration field.

The diagram delineates the multifaceted applications of piezoelectric biomaterials within the medical domains of pathogenic eradication and tissue regeneration. Pathogenic eradication involves antibacterial actions against free bacteria and biofilms, as well as the treatment of soft tissue infections, implant infections and osteomyelitis and biofilm fouling. Moreover, tissue regeneration involves the employment of piezoelectric biomaterials in the regenerations of skin, bone, cartilage, and nerve. Collectively, these applications underscore the expansive potential of piezoelectric biomaterials in enhancing healthcare outcomes.

## 2 Piezoelectric Biomaterials

Traditional piezoelectric materials can be classified into three types on the basis of their crystal structure and chemical composition: ionic, atomic and molecular crystal structures. Traditional piezoelectric materials are represented by ionic crystals such as PZT. Their piezoelectric coefficient is high, but some lead‐containing ceramics have problems with biological toxicity and environmental pollution. They are mainly used in industrial fields, such as acoustic devices, precision drives, and energy collection. In contrast to traditional piezoelectric materials, piezoelectric biomaterials prioritize biocompatibility, degradability, and safety for in vivo applications [8]. These include both organic and inorganic systems that exhibit intrinsic or engineered piezoelectricity [9]. On the organic side, natural polymers such as collagen, chitin, and silk fibroin, as well as synthetic polymers like polyvinylidene fluoride (PVDF) and its copolymers, offer excellent flexibility, low cytotoxicity, and adaptability to soft tissues, making them ideal for neural interfaces, tissue scaffolds, and implantable biosensors, despite their relatively lower piezoelectric coefficients compared to ceramics [10]. Meanwhile, several biocompatible inorganic materials have also shown great promise in biomedical applications. Lead‐free ceramics such as BTO support cell adhesion and osteogenic differentiation, while ZnO nanostructures exhibit both piezoelectric and antibacterial properties, and can promote angiogenesis. In addition, hydroxyapatite‐based piezoelectric composites, which mimic native bone mineral, have been investigated for bone tissue engineering. These inorganic materials offer tunable electrical output, chemical stability, and biological compatibility, making them well‐suited for implantable devices and electroactive tissue engineering platforms.

### 2.1 Structure Categorization of Piezoelectric Materials

From the perspective of their structure, current piezoelectric materials can be classified into three categories: inorganic piezoelectrics, organic piezoelectrics, and piezoelectric composites (Table 1). Inorganic piezoelectrics, such as lead‐free ceramics like BTO and ZnO, are characterized by robust piezoelectric coefficients and chemical stability. Organic piezoelectrics, including polymers like polyvinylidene fluoride (PVDF) and poly‐l‐lactic acid (PLLA), offer greater flexibility, low toxicity, and ease of processing. Piezoelectric composites, which integrate inorganic and organic phases, are designed to combine the superior piezoelectric response of ceramics with the mechanical compliance and biocompatibility of polymers. Notably, a subset of these materials have demonstrated excellent biological compatibility and are increasingly being explored for biomedical applications.

**TABLE 1:** Representative piezoelectric materials classified by their structure.

Material class	Structural type	Representative materials
Inorganic piezoelectrics	Perovskite	PZT, BTO, KNbO_3_
	Wurtzite	ZnO, AlN, CdS
	Lithium niobate type	LiNbO_3_, LiTaO_3_
	Tungsten bronze	Sr_x_Ba_1‐x_Nb_2_O_6_, Ba_2_NaNb_5_O_15_
Organic piezoelectrics	Polar crystal	PA11, PLLA, β‐glycine, and γ‐glycine
	Semicrystalline	β‐PVDF, PVDF‐TrFE
	Amorphous polymer	Vinylidene dicyanide/vinyl acetate copolymer
Piezoelectric composites	0‐3 connectivity	BTO‐silicone composite, BTO‐PVDF composites, ZnO‐PLLA composites
	1‐3 connectivity	PMN‐PT single crystal fiber/polyurethane composite, PZT rods/polymer
	2‐2 connectivity	Aluminum nitride/silicon heterojunction, PZT thin film/alumina multilayer array

**Abbreviations**: PA11, polyamide 11; PLLA, β‐polylactide; PVDF‐TrFE, poly(vinylidene fluoride‐cotrifluoroethylene); β‐PVDF, β‐poly(vinylidene fluoride).

The piezoelectric properties of piezoelectric ceramics are influenced by their crystal structures. Perovskite structures, found in materials like PZT, are used in applications from energy harvesting to sensing. However, lead component limits its biological use, prompting the development of lead‐free alternatives like BTO, known for good electromechanical coupling and biocompatibility [11]. Wurtzite structures, present in materials such as ZnO and aluminum nitride (AlN), are utilized in imaging systems and sensors due to their piezoelectric qualities and biocompatibility [12, 13]. Although piezoelectric ceramics offer many advantages, traditional piezoelectric ceramics suffer from excessive hardness, brittleness, and non‐biodegradability. To further improve their applicability in biomedical contexts, piezoelectric ceramics are often combined with polymers or incorporated into composite systems to achieve a balance between piezoelectric performance and mechanical compliance [14]. For instance, nanostructured BTO or ZnO particles can be embedded in biodegradable polymer matrices to fabricate flexible, biocompatible piezoelectric scaffolds or sensors. These composite materials not only retain the functional properties of their ceramic components but also exhibit improved processability, stretchability, and tissue‐level integration. Additionally, recent advances in microfabrication techniques have enabled the design of patterned piezoceramic thin films with tunable geometries and surface properties, further enhancing their performance in implantable devices and dynamic tissue environments.

Piezoelectric polymers, owing to their asymmetric molecular structures, exhibit ferroelectricity and piezoelectricity, making them suitable for applications in flexible, implantable electronic devices because of their mechanical flexibility, light weight, and biodegradability [15, 16, 17]. PVDF, a prominent piezoelectric polymer, achieves optimal piezoelectric properties when in its β‐phase and is further enhanced in performance through copolymers like P(VDF‐TrFE), which offer superior crystallinity and stability in biological environments [18, 19, 20]. Other polymers such as poly‐l‐lactic acid (PLLA) and polyvinyl chloride (PCL) also display piezoelectric effects, contributing to their potential in biomedical applications [21]. Despite their lower piezoelectric charge coefficients compared to inorganic materials, piezoelectric polymers can be combined with inorganic components to create composites that enhance piezoelectricity, biocompatibility, and mechanical flexibility, offering a versatile alternative for various biomedical applications [22].

### 2.2 Classification of Piezoelectric Biomaterials Based on Material Origins

From the perspective of material origin, piezoelectric biomaterials can be broadly classified into natural and synthetic categories. Natural piezoelectric biomaterials are derived from biological tissues or naturally occurring products and are distinguished by their excellent biocompatibility and biodegradability, although their piezoelectric coefficients are generally lower than those of synthetic counterparts. Biomolecular piezoelectric materials, including amino acids, peptides, proteins, and biological tissues such as bone and tendon, exhibit piezoelectric properties due to their inherently asymmetric molecular structures. This structural asymmetry enables these materials to convert mechanical stress into electrical signals, a capability with far‐reaching implications in biomedical engineering and sensor technologies [23, 24, 25]. Natural polymers such as collagen, chitosan, glycine, and silk fibroin are particularly notable; their unique molecular configurations, such as collagen's triple‐helix structure, endow them with intrinsic piezoelectricity, making them highly suitable for applications in tissue engineering scaffolds, wound healing, and neural regeneration [26, 27]. Similarly, amino acids like β‐glycine and γ‐glycine demonstrate piezoelectricity as a result of their non‐centrosymmetric crystal structures, while collagen's triple‐helix also confers piezoelectric functionality [28, 29, 30]. These materials’ ability to transduce mechanical signals into biological responses is critical for regulating cellular activities involved in tissue maintenance and regeneration. In summary, the inherent piezoelectricity of these biomolecules and tissues opens promising avenues for regenerative medicine, especially in the development of scaffolds that provide electrical cues to enhance cellular activity and differentiation.

In contrast, synthetic piezoelectric biomaterials, obtained through chemical synthesis or artificial fabrication, typically offer higher and tunable piezoelectric performance along with structural flexibility. Prominent examples include piezoelectric ceramics such as BTO and ZnO, as well as piezoelectric polymers like PVDF, its copolymers, and PLLA. These materials can be engineered with precise control over their crystallinity, morphology, and composition, allowing for the optimization of their electrical and mechanical properties to suit specific biomedical applications. Synthetic piezoelectric materials are widely employed in the development of flexible sensors, implantable electronics, smart wearable devices, and energy‐harvesting platforms. Furthermore, the combination of synthetic piezoelectric materials with biocompatible polymers or hydrogels enables the fabrication of composite systems that integrate robust piezoelectric performance with enhanced processability, flexibility, and biological compatibility. Such composites are increasingly used in tissue engineering scaffolds, bioactive wound dressings, and advanced neural interfaces, expanding the functional versatility and clinical potential of piezoelectric biomaterials in regenerative medicine and biomedical engineering.

### 2.3 Classification of Piezoelectric Nanomaterials Based on Morphology

Advances in nanotechnology provide new paradigms for the development of piezoelectric nanomaterials. Overall, piezoelectric nanomaterials have merits in biomedical applications in comparison to their bulk counterparts. The ultra‐small size of piezoelectric nanomaterials allows them to effectively cross various physiological barriers, such as blood vessels or cell membranes. In addition, for biomedical applications based on piezoelectric catalysis, piezoelectric nanocatalysts typically exhibit better catalytic efficiency than bulk catalysts because their smaller size provides higher electron transfer rates and stronger interactions with any substrate. Moreover, the high surface area of piezoelectric nanomaterials enables them to serve as multifunctional drug nanocarriers for drug delivery and disease treatment. Therefore, bio piezoelectric nanomaterials with piezoelectric properties and nanoscale effects have broad biomedical application potential. In general, piezoelectric nanomaterials can be classified into three categories according to their shapes, including piezoelectric nanoparticles, one‐dimensional nanomaterials and two‐dimensional nanosheets.

#### 2.3.1 Piezoelectric Nanoparticles

Piezoelectric nanoparticles are characterized by their large surface area, impressive piezoelectric properties, and uniform single‐domain structure [31]. Inorganic piezoelectric materials such as PZT, BTO, BN, and ZnO often outperform their organic counterparts, mainly due to their exceptionally high piezoelectric coefficients and strong mechanical properties. Specifically, PZT nanoparticles possess high piezoelectric coefficients, making them suitable for energy harvesting and sensing applications in the biomedical field. However, the presence of lead raises concerns regarding their toxicity, which limits their direct use in vivo. Thus, researchers prefer to develop lead‐free alternatives or modify PZT nanoparticles to reduce the leaching of lead while maintaining their piezoelectric performance [32]. BTO nanoparticles are notable for their high biocompatibility and rapid metabolic rates, making them especially suitable for applications in the biomedical field. These features ensure that such materials can meet the stringent demands of medical applications where both functionality and biocompatibility are critical [33]. BTO nanoparticles have been explored for various biomedical applications. Their piezoelectric properties can induce electrical signals that may enhance cell behavior, such as proliferation and differentiation [34]. For example, in bone tissue engineering, the piezoelectric effect of BTO nanoparticles incorporated into scaffolds can mimic the natural piezoelectricity of bone and potentially promote osteoblast activity. Organic piezoelectric nanoparticles contain PVDF and its copolymers nanoparticles and cellulose nanocrystals (CNCs) [35]. PVDF is a well‐known piezoelectric polymer. When fabricated into nanoparticles, it shows promise in biomedical devices. For instance, PVDF nanoparticles have been used in wearable and implantable sensors. Their flexibility and biocompatibility make them suitable for applications where conformal contact with biological tissues is required. The piezoelectric response of PVDF nanoparticles can be tuned by various methods such as copolymerization and mechanical stretching, allowing for customization according to specific application needs. In addition, piezoelectric nanoparticles can integrate with other piezoelectric biomaterials such as polymer films to improve their piezoelectric performance. Derived from natural cellulose, CNCs with piezoelectricity have emerged as a sustainable option. They can be incorporated into composites for biosensing applications. The unique combination of piezoelectricity and the biodegradable nature of CNCs makes them attractive for use in temporary medical implants or drug delivery systems where the device needs to degrade over time without causing harm to the body. Inorganic–organic hybrid nanoparticles combine the advantages of both inorganic and organic materials. For example, a hybrid nanoparticle composed of BTO core coated with a layer of PVDF can offer enhanced piezoelectric performance and better biocompatibility compared to the individual components. The inorganic core provides high piezoelectricity, while the organic coating can improve the interaction with biological systems [36]. Such hybrid nanoparticles are being investigated for applications in regenerative medicine and bioelectronics, where the combination of properties is crucial for effective device function. Thus, the advantages of piezoelectric nanoparticles enable them to be employed in a number of versatile biomedical applications, such as biocatalysis and disease treatment [37].

#### 2.3.2 Piezoelectric One‐Dimensional Nanomaterials

One‐dimensional (1D) piezoelectric nanomaterials, such as nanowires, nanobelts, nanotubes, nanorods, and nanofibers, stand out due to their wise‐like morphology, which enhances charge transfer efficiency compared to piezoelectric nanoparticles [38]. This structure also helps to mitigate the issue of agglomeration often seen with nanoparticles, leading to improved processability, enhanced piezoelectric effects, higher sensitivity, and greater flexibility [39, 40]. Notably, most 1D piezoelectric nanomaterials are highly biocompatible, making them ideal for use in a variety of applications including biosensors, smart textiles, and electronic skins [41, 42]. ZnO is one of the most extensively studied inorganic piezoelectric materials for nanowire applications. It has a relatively simple synthesis process and exhibits excellent piezoelectric properties. These nanowires can be grown on various substrates and have been used in biosensing applications. For example, in detecting biomolecules, the piezoelectric effect of ZnO nanowires can be utilized to transduce the binding events of biomolecules into electrical signals [43]. They also show potential in energy harvesting for implantable biomedical devices, where the mechanical deformations in the body can cause the nanowires to generate electricity. TiO_2_ nanowires possess good chemical stability and biocompatibility, they can be functionalized for specific bio‐interactions, such as in biosensors for detecting biomarkers related to diseases [44]. In addition, some organic piezoelectric, such as polylactic acid (PLA) nanowires [45], have been used in flexible and wearable biomedical devices.

#### 2.3.3 Piezoelectric Two‐Dimensional Nanosheets

When reduced to nanometer thickness, piezoelectric materials, and even some traditionally non‐piezoelectric materials can lose their centrosymmetry in one direction, leading to enhanced piezoelectric properties [31, 46]. This transformation enables the creation of 2D piezoelectric nanomaterials with varied planar structures such as nanoplatelets, nanoplates, nanosheets, and nanoflowers. Prominent examples of these 2D piezoelectric nanomaterials include black phosphorus [47], boron nitride [48], carbon nitride [49], and monolayer transition metal dihalide dichalcogenides [50]. These materials demonstrate significant potential due to their unique properties which are leveraged in various high‐tech applications. In particular, materials like MoS_2_ have demonstrated piezoelectric properties when reduced to an odd number of layers, while even‐layered MoS_2_ sheets do not exhibit these properties. This indicates that the piezoelectricity in these materials is highly dependent on their atomic layer configuration, which can significantly impact their electronic, optical, and mechanical properties [51]. This behavior also extends to other transition metal dichalcogenides and insulating monolayer materials, such as hexagonal boron nitride. These 2D nanomaterials, particularly in their ultrathin form, open up a wide array of possibilities for applications ranging from drug delivery systems to energy harvesting and sensing technologies due to their unique piezoelectric, flexoelectric, and electromechanical characteristics.

## 3 Antibacterial Mechanism of Piezoelectric Biomaterials

The piezoelectric effect arises from the displacement of positive and negative charge centers (i.e., formation of electric dipoles) in piezoelectric materials under mechanical stress due to their non‐centrosymmetric crystal structure [52, 53]. In addition, ultrasonic waves provide a controllable mechanism for applying compressive stress to piezoelectric materials, generating localized electric fields through the piezoelectric effect. This field‐driven polarization facilitates charge carrier separation at the material interface, creating transient electric dipoles that enhance interfacial redox activity [54]. The electric dipoles endow piezoelectric materials with robust catalytic properties [55]. Both piezoelectricity and piezocatalysis can exert profound impacts on pathogens and their microenvironment, with potential mechanisms including catalyzing ROS generation, disrupting biofilm architecture, and disrupting bacterial membrane permeability and metabolic processes, among others.

### 3.1 Piezoelectric Catalysis for ROS Generation

**FIGURE 2:**
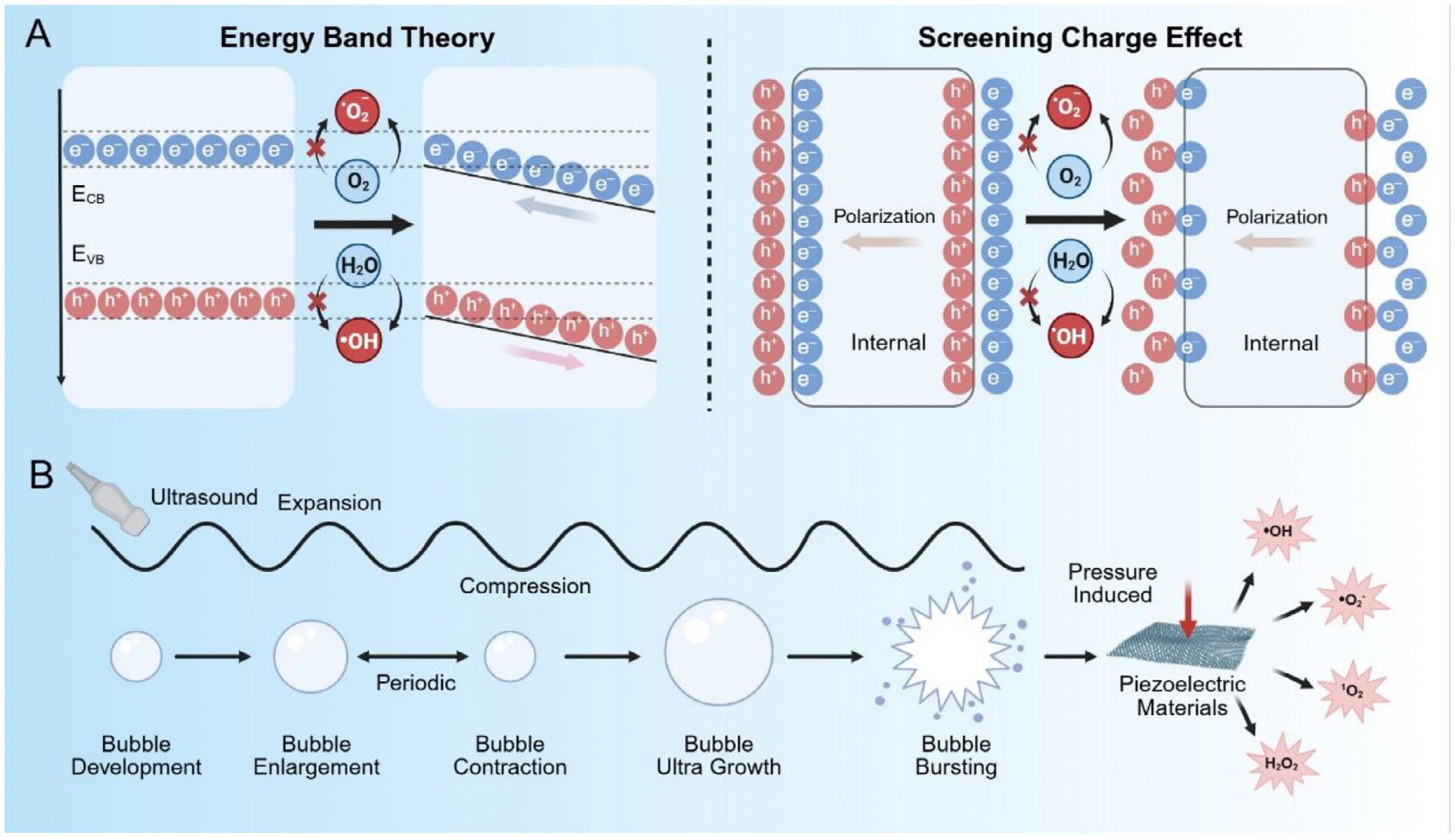
Schematic diagram of piezoelectric catalysis. (A) Two popular theories for the explanation of piezocatalysis. Energy band theory: Piezoelectric deformation induces band bending that promotes electron–hole separation and enhances redox activity at catalytic sites; screening charge effect: piezoelectric polarization attracts mobile charges to catalyst surfaces, forming high‐density reaction zones that drive interfacial redox processes. (B) Diagram of ultrasonic activated piezoelectric effects.

Piezoelectric materials, typically noncentral symmetric crystals, generate voltage under pressure across their opposing end faces [56]. They are capable of converting mechanical force into an electrical field and vice versa through the piezoelectric effect, leading to the accumulation of negative and positive charges on their opposite surfaces [57]. In piezocatalysis, this property facilitates the generation of charge carriers when externally stimulated, followed by oxidation‐reduction reactions on the material's surface [58]. These reactions transform relatively inert molecules like oxygen (O_2_) and water (H_2_O) into highly ROS such as superoxide free radicals (·O_2_), singlet oxygen (^1^O_2_), hydroxyl radicals (·OH), and hydrogen peroxide (H_2_O_2_). Two predominant theoretical frameworks currently explain piezoelectric catalysis: energy band theory and screening charge effect (Figure 2A). The energy band theory postulates that the piezoelectric effect establishes a built‐in electric field, which modifies the potential of valence band (VB) and conduction band (CB) through band bending. This structural deformation facilitates enhanced electron–hole separation and elevated redox reactivity at active sites. Conversely, the screening charge effect posits piezoelectric potential as the primary driver. The polarized surfaces attract mobile screening charges through Coulombic interactions, creating concentrated redox‐active zones where vigorous interfacial reactions occur between the accumulated charges and surrounding reactants.

To date, ROS‐mediated therapeutic strategies have been well developed, including enzymatic catalytic therapy [59], sonodynamic therapy (SDT) [60, 61], photocatalytic therapy [62, 63, 64], photodynamic therapy (PDT) [37], and chemodynamic therapy (CDT) [65]. Among them, SDT demonstrates compelling advantages due to exceptional penetration capability of US in biological tissues, allowing for non‐invasive focusing of acoustic energy on deep‐seated tissues while activating sonosensitizers to generate ROS The emergence of piezoelectric catalysis has driven the advancement of SDT, with the US employed in SDT representing the most common external mechanical force for activating piezoelectric biomaterials [66, 67]. In 2010, Hong et al. demonstrated the ability of piezoelectric materials (such as BTO and ZnO) to catalyze water spitting into H_2_ and O_2_ in response to US stimulation [68]. The process involves piezoelectric materials deforming under external force, generating a strain‐induced electromotive force on their surfaces, which then transfers charges to water molecules. The US‐driven piezocatalytic mechanism initiates with cavitation dynamics in aqueous environments, where alternating compression‐rarefaction cycles generate oscillating vapor‐filled microbubbles [2]. During bubble collapse phases, these cavities implode, producing high‐velocity microjets that impact piezoelectric surfaces with transient pressures (Figure 2B). This mechanical force induces lattice deformation in the piezoelectric material, establishing localized potential gradients through strain‐mediated polarization [69]. The strength of the internal electric field driving carrier migration is crucial for the efficacy of piezoelectric catalysis and is influenced by the material's dielectric constant and ultrasound energy input. To enable effective therapy under low‐intensity ultrasound and minimize damage to healthy tissue, Chen et al. designed a piezoelectric bio‐heterojunction (P‐bioHJ) composed of bismuth oxyiodide (BiOI) and few‐layer MXene [70]. This structure, with a narrow band gap responsive to sonoluminescence, promotes interfacial polarization and surface oxygen vacancy formation. These vacancies enhance BiOI's piezoelectricity, facilitate charge separation by acting as electron traps, improve oxygen intermediate adsorption, and reduce the energy barrier for radical generation, collectively enabling rapid and efficient antibacterial activity.

### 3.2 Interaction With Biofilm Architecture

Bacterial biofilm infections represent the predominant form of most microbial infections, characterized as bacterial communities encased within extracellular macromolecules and possessing defined three‐dimensional architectures and functional capabilities. The extracellular polymeric substances (EPS) matrix functions as a physical barrier, significantly augmenting bacterial antimicrobial resistance [71]. Therefore, the rapid biofilm‐penetrating capacity of piezoelectric antibacterial materials stands as another critical advantage in combating such infections.

When mechanical stimulation is applied, piezoelectric materials can generate surface charges. Zhao et al. developed a PVDF‐TrFE piezoelectric nanofiber film (PNF) [72]. The periodic fluctuating stress induced by US stimulation disrupts the equilibrium between bound charges and screening charges. Excess screening charges are released from the PNF surface and transferred into the biofilm. Since EPS inherently possess electrochemical activity, the piezoelectric treatment can promote charge transfer kinetics to disrupt the molecular structure of bacterial EPS, thereby enhancing the antibacterial activity of therapeutic agents.

Moreover, the presence of biofilm formation compromises macrophage phagocytosis and even induces polarization of macrophages toward the anti‐inflammatory M2 phenotype, resulting in immune evasion by pathogens [73]. Piezoelectric biomaterials exert combined therapeutic effects by mechanically disrupting biofilm structures to expose embedded pathogens, which subsequently activate immune cells and trigger pro‐inflammatory cytokine release. Concurrently, ROS generated through piezoelectric stimulation further amplify inflammatory responses, establishing a self‐reinforcing antimicrobial cycle that simultaneously degrades biofilm matrix and enhances host immunity. For example, Li et al. constructed a piezoelectric layer on the surface of titanium implants by forming BTO nanostructures in situ on Ti, followed by depositing gold nanoparticles as co‐catalysts [74]. When the implant is infected by biofilms, US‐induced mechanical stress activates the piezoelectric effect, which downregulates biofilm‐related genes and activates the phosphoinositide 3‐kinase‐protein kinase B pathway (PI3K‐AKT) and mitogen activated protein kinase (MAPK) inflammatory pathways in macrophages, thereby enhancing bacterial phagocytosis.

### 3.3 Disrupting Bacterial Membrane Permeability and Metabolic Processes

The phosphate groups and carboxyl functional groups on the bacterial surface impart a negative charge to the bacterial cell membrane. Consequently, bacteria are readily attracted to the “positive pole” of piezoelectric materials. Subsequent charge neutralization alters the permeability of the bacterial cell membrane, and the underlying mechanism may involve Coulombic interaction forces [75, 76]. This antimicrobial mechanism may function independently of ROS, considering that specific piezoelectric polymers (e.g., PLLA and PVDF) demonstrate inadequate piezoelectric responses to induce water hydrolysis for ROS production. Gazvoda et al. fabricated fully organic piezoelectric biodegradable films composed of PLLA, demonstrating that the positively charged surface of piezoelectric films generates endogenous electric fields with bacterial membranes [21]. This interaction triggered membrane damage and disruption of transmembrane potential, subsequently compromising normal cellular morphology and ultimately leading to bacterial death. Notably, their research substantiated that pH fluctuations and ROS generation made negligible contributions to this antibacterial mechanism.

In addition, the electric potential generated by piezoelectric materials can disrupt the electron transport chain (ETC) of pathogens and interfere with transmembrane substance transport processes in bacterial membranes, thereby effectively eliminating the pathogens. For example, Geng et al. investigated the inhibitory effects of piezoelectric heterojunction materials on bacterial metabolism [77]. The results revealed that the piezoelectric properties significantly impaired the activity of Complex IV (a key component in the electron transport chain), disrupted electron flow, obstructed ATP synthesis, and ultimately caused substantial suppression of bacterial ATP production, thereby exhibiting bactericidal efficacy.

## 4 Possible Regeneration Mechanism of Piezoelectric Materials

Bioelectricity is fundamental to a host of biological functions, influencing key processes from embryonic development to tissue repair [5]. It operates through endogenous electric fields (EFs) that regulate critical cellular processes such as chemotaxis, migration, proliferation, differentiation, cell division, and intracellular communication [78]. These electric fields are also integral to neuronal activities, mechanotransduction, ion transport, and the healing of bone and epithelial tissues. Given the pivotal role of bioelectricity, various therapeutic applications have been developed, such as electrotherapy. This approach employs external devices that deliver low‐level electric currents across the skin to promote wound healing, stimulate deep brain activities, support tissue regeneration, and aid recovery in musculoskeletal conditions and bone fractures. However, the use of these external devices can often be cumbersome and uncomfortable for patients.

To address these challenges, research has shifted towards developing piezoelectric biomaterials that naturally generate electric signals in response to mechanical changes such as cell movement, body dynamics, or external forces like US and vibration. These biomaterials possess the unique capability to convert mechanical energy into electrical energy and vice versa, known as reverse piezoelectricity. This transformation enables the direct and effective integration of electrical stimuli into physiological processes, offering a seamless, less invasive alternative to traditional electrotherapy devices in medical applications. Piezoelectric effect is a special type of electrical stimulation, due to electric fields that can affect the development and regeneration of tissues, the built‐in electric field induced by mechanical force allows piezoelectric materials to be widely used in tissue repair and engineering [4]. Below, several possible mechanisms will be summarized.

### 4.1 Electrical Signal

**FIGURE 3:**
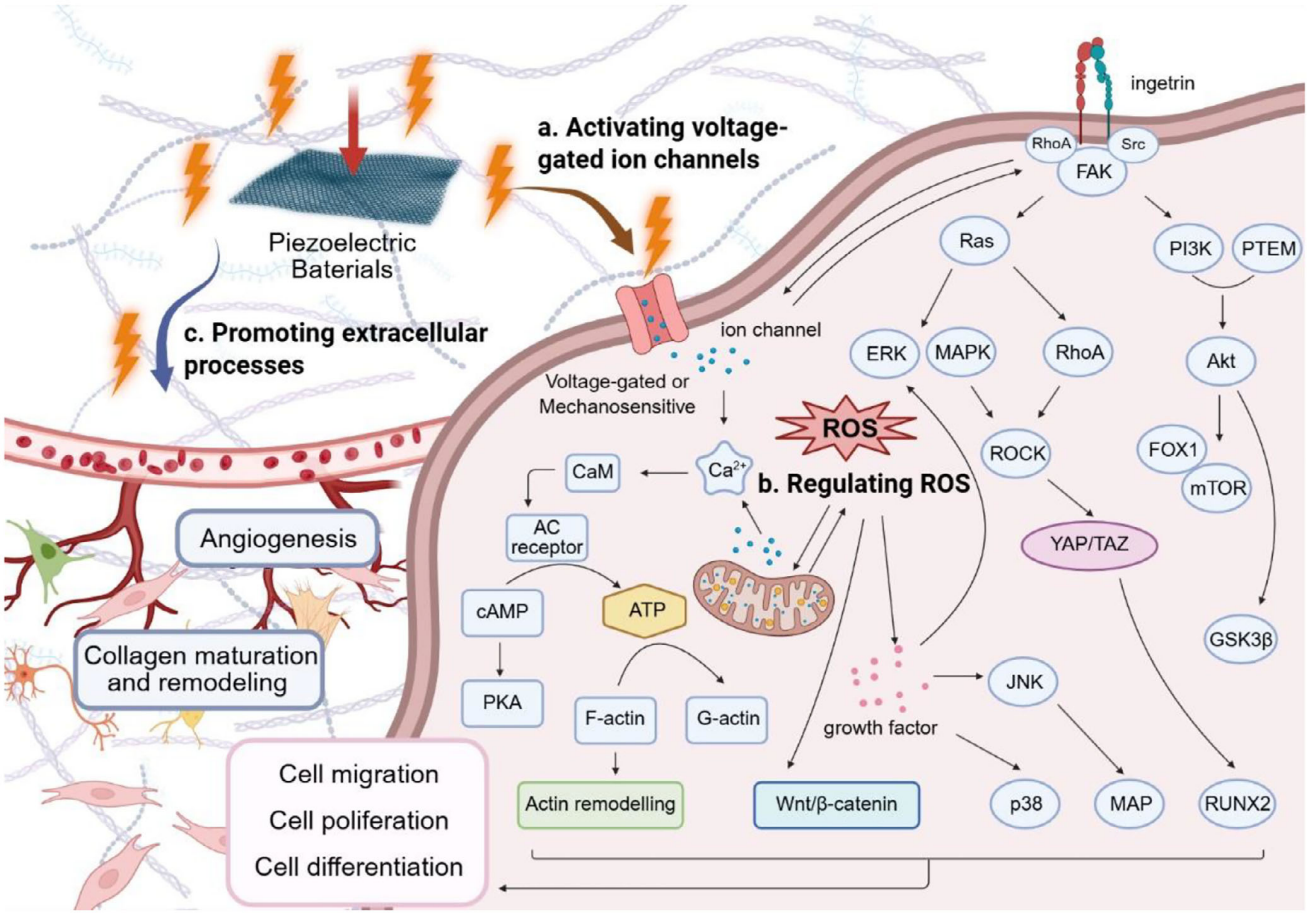
Electrical signal mechanisms of piezoelectric biomaterial‐mediated tissue regeneration. (a) Piezoelectric materials generate local electric fields under mechanical stress, activating voltage‐gated calcium channels (VGCCs) and promoting Ca^2+^ influx, which initiates osteogenic signaling pathways (e.g., Wnt/β‐catenin, PI3K/Akt, NFAT) and upregulates osteogenic markers. (b) Piezoelectric stimulation regulates intracellular ROS and growth factor expression, activating pathways, such as ERK/MAPK and RhoA–ROCK–YAP/TAZ, to regulate proliferation, migration, and differentiation. (c) Extracellularly, piezoelectric cues enhance angiogenesis and collagen remodeling, supporting functional tissue regeneration. Abbreviation: CaM, calmodulin; AC, adenylyl cyclase; cAMP, cyclic adenosine monophosphate; PKA, protein kinase A; ERK, extracellular signal regulated kinase; FAK, focal adhesion kinase; JNK, c‐jun N‐terminal kinase; MAPK, mitogen activated protein kinase; PI3K, phosphatidylinositol‐3 kinase; PTEN, phosphate and tensin homolog; Src, steroid receptor coactivator; ROCK, Rho‐associated protein kinase; FOX1: forkhead box protein 1; mTOR, mammalian target of rapamycin; GSK3β, glycogen synthase kinase‐3.

Ion channels are transmembrane protein structures embedded within the lipid bilayer of the cell membrane, forming hydrophilic pores that allow the selective passage of specific ions such as Na^+^, Ca^2+^, and K^+^. The ionic concentration gradient across the membrane gives rise to a membrane potential, which plays a fundamental role in regulating ion exchange and electrical signal transmission between cells. Endogenous bioelectrical signals dynamically modulate this membrane potential, thereby influencing cellular excitability and intercellular communication. Piezoelectric biomaterials, when subjected to mechanical stress, generate localized electric fields via the piezoelectric effect. These surface charges can activate voltage‐gated ion channels, such as voltage‐gated calcium channels (VGCCs), on the cell membrane, leading to an influx of Ca^2+^ ions (Figure 3a). This rise in intracellular calcium concentration serves as a key biochemical signal, especially in promoting osteogenic differentiation and mineralization in stem cells. Notably, VGCC family members such as Cav1.2 and Cav3.1 have been reported to be upregulated in response to electrical stimulation, thereby activating downstream signaling pathways including Wnt/β‐catenin, PI3K/Akt, and the calcineurin/NFAT axis [63, 79]. These cascades ultimately enhance the expression of osteogenic markers such as osterix (OSX), RUNX2, alkaline phosphatase (ALP), osteopontin (OPN), and osteocalcin (OCN) [80]. For instance, Mao et al. demonstrated that the surface charge characteristics of a BaTiO_3_/β‐TCP (BTCP) scaffold influence cell fate: negatively charged surfaces enhanced Ca^2+^ influx and promoted the osteogenic differentiation of bone marrow stem cells (BMSCs), while positively charged surfaces favored M2 macrophage polarization, contributing to a regenerative immune microenvironment [81]. Within the cell, Ca^2+^ binds to calmodulin (CaM), a key calcium‐sensing protein. Upon activation, CaM interacts with and activates a range of target proteins that do not directly bind calcium themselves, thus initiating various downstream regulatory pathways. One such pathway involves the CaM‐mediated activation of adenylate cyclase, which increases intracellular cyclic AMP (cAMP) levels and has been implicated in neurogenesis and stem cell function via the cAMP/PKA signaling axis [82, 83]. Beyond calcium signaling, piezoelectric stimulation can regulate intracellular levels of ROS and enhance the expression of various growth factors (Figure 3b). These secondary messengers activate multiple regenerative pathways, including ERK/MAPK, PI3K/Akt, JNK, and RhoA–ROCK–YAP/TAZ, which regulate gene expression and influence cell migration, proliferation, and differentiation. The role of ROS in tissue regeneration is notably dose‐dependent: physiological levels of ROS serve beneficial signaling roles in processes such as wound healing, immune modulation, and stem cell activation, whereas excessive ROS accumulation can induce oxidative stress, impair cell viability, and disrupt regenerative outcomes [84]. Therefore, precise modulation of ROS levels is critical in piezoelectric biomaterial‐based strategies to ensure they remain within a therapeutic window. In addition to these intracellular effects, piezoelectric stimulation can promote extracellular processes, including angiogenesis and collagen maturation/remodeling, both of which are essential for robust tissue regeneration (Figure 3c). Altogether, these interconnected mechanobiological and biochemical mechanisms highlight the multifaceted role of piezoelectric biomaterials in coordinating regenerative events across molecular, cellular, and tissue levels.

### 4.2 Mechanical Signal

Cells attached to the surface of piezoelectric materials can receive mechanical signals from the material surface (such as the material's geometry, stiffness, and mechanical forces generated by compression and tension) and convert them into biological signals, thereby regulating the physiological activities of cells [85]. In the cell differentiation regulatory system, a key player in stretch activation is the Piezo protein family, which consists of two isoforms: Piezo1 and Piezo2 [86]. They exhibit special mechanical sensitivity and can respond to changes in membrane tension, functioning as mechanically sensitive cation channels [87]. Piezo2 is involved in mediating light touch, while Piezo1 plays a more critical role in the process of cell differentiation regulated by mechanical stress. Piezo1, a mechanically sensitive calcium‐permeable channel, is a nonselective cation‐conducting channel with a slight preference for Ca^2+^[88]. Piezo1 channels, sensitive to mechanical forces such as fluid shear stress and extracellular matrix stiffness, initiate signaling cascades involving dephosphorylation of NFATc1, YAP1, and β‐catenin, essential for bone formation and mineralization [89]. Conversely, the absence of Piezo1 leads to severe bone defects, underscoring its role in bone homeostasis, with periosteum macrophages also responding to mechanical loading via Piezo1 to facilitate bone formation [90].

Cells can regulate cellular behavior by activating intracellular signaling pathways through ligand‐induced extracellular surface receptors in response to the perception of extracellular adhesion nanoforms [91, 92]. Ligand‐receptor signaling pathways such as Notch, Wnt, and transforming growth factor‐beta (TGF‐β) play critical roles in bone formation and are influenced by mechanical and electrical stimuli provided by piezoelectric materials. The Notch pathway, activated by mechanical stress, involves the interaction between the Notch receptor and a ligand from a neighboring cell, triggering a cascade that results in gene transcription crucial for stem cell differentiation and bone regeneration [93, 94]. Similarly, Wnt signaling, integral to various physiological processes, is activated by mechanical loading which inhibits glycogen synthase kinase‐3 beta (GSK‐3β), leading to the activation of genes involved in osteogenesis. This pathway is particularly responsive to both compressive forces and electrical stimuli, which can enhance osteogenesis [95]. The TGF‐β pathway, mediated by type I and II kinase receptors, also plays a significant role in bone formation, responding to mechanical forces and electrical fields, which synergize with bone morphogenetic protein‐2 (BMP‐2) to promote bone formation [96]. Mechanical forces can further enhance this pathway's activity through macrophages detecting mechanical loading via the Piezo1 ion channel, activating TGF‐β1 and influencing bone formation processes. These pathways illustrate how mechanical and electrical inputs via piezoelectric materials can be harnessed to enhance cellular communication and osteogenesis, making them potent tools in regenerative medicine.

### 4.3 Other Possible Mechanisms

Wound healing typically involves three overlapping phases: the inflammatory phase, the proliferative phase, and the remodeling phase. Emerging evidence suggests that electrical stimulation generated by piezoelectric materials can positively influence all three stages. In the early inflammatory phase, electrical cues promote vasodilation, enhance vascular permeability, and improve local blood perfusion. They also activate endothelial cells near the injury site, stimulating neovascularization and facilitating vascular infiltration into poorly perfused areas, thereby restoring blood supply to damaged tissues. Studies have demonstrated that direct current stimulation not only enhances angiogenesis but also modulates the expression of key growth factors and cytokines through signaling pathways mediated by vascular endothelial growth factor (VEGF) [97]. In the proliferative phase, electrical stimulation has been shown to accelerate wound contraction, fibroblast proliferation, angiogenesis, and collagen deposition. During the remodeling phase, it facilitates the maturation and reorganization of collagen fibers, contributing to faster and more effective wound closure by reducing wound area and improving tissue integrity [98]. Thus, piezoelectric biomaterials can generate bioelectric signals in response to mechanical stimuli, thereby actively participating in and promoting the wound repair process.

## 5 Strategies for Optimizing Piezoelectric Properties

Despite the inherent high catalytic efficiency of nano‐sized piezoelectric catalysts, attributed to their extensive specific surface area, the catalytic performance of current piezoelectric catalysts remains suboptimal. One significant limitation is that in piezoelectric nanoparticles featuring multiple ferroelectric domains, transient piezo‐induced electrons and holes are quickly consumed, limiting the availability of these charges for redox reactions [99]. Additionally, pristine piezoelectric catalysts often exhibit low surface work functions, making them less effective for certain molecular redox reactions [100]. To overcome these challenges, there is a pressing need for the efficient separation of piezo‐induced charges and the development of an active catalytic interface. This can be achieved through targeted surface modification and engineering of piezoelectric materials to enhance their nanoscale piezocatalytic performance and meet specific biomedical requirements [101]. At present, the methods used to improve the piezoelectric performance mainly include adjusting the surface characteristics, constructing defects, introducing dopants and forming composites.

### 5.1 Refinement Crystal Grain Size and Morphology

Enhancing the piezoelectric efficiency of inorganic piezoelectric biomaterials can be effectively achieved through the refinement of crystal grain size and control of material morphology. Among various strategies, tuning the grain size has been shown to exert a direct influence on domain structure and polarization behavior. For example, optimizing the grain size of BTO ceramics to approximately 1 µm leads to a substantial enhancement in piezoelectric performance, yielding a peak piezoelectric coefficient of 519 pC N^−1^ and a maximum dielectric constant of 6079 [102]. This improvement is attributed to an optimal balance between grain boundary density and domain mobility, which facilitates more efficient dipole alignment under external stimuli. In parallel, morphological engineering of piezoelectric nanomaterials, such as tailoring aspect ratios, introducing porosity, or constructing core–shell architectures, further influences their piezoelectric behavior. Variations in morphology can significantly affect the surface area‐to‐volume ratio and alter the local dipole distribution, thereby modulating both short‐ and long‐range dipolar ordering [103]. Given the intrinsic coupling between mechanical properties and geometric structure [104], rational design of nanomaterial morphology offers a promising avenue to amplify electromechanical coupling. Together, precise control over both grain size and morphology provides a promising method for maximizing the piezoelectric response of inorganic biomaterials in biomedical applications.

### 5.2 Construction of Defects

Defects are ubiquitous in materials and have a significant impact on their properties. Vacancy engineering can regulate the electronic structure of piezoelectric materials through generating vacancy defects to increase the charge carrier concentration [105]. Oxygen vacancies (OVs) in piezoelectric materials play a critical and dual role in influencing their catalytic properties [106, 107]. OVs can enhance piezoelectric catalytic efficiency by introducing defective energy levels that reduce the energy required for carrier generation, thereby increasing carrier concentration [108]. They also enhance the adsorption of molecules like O_2_ and OH^−^, boosting the production of ROS. Conversely, OVs may reduce piezoelectricity by impairing carrier separation and migration efficiency [109]. He et al. managed to control the concentration of OVs by adjusting the thermal reduction temperature using NaBH_4_ [110]. Their findings showed that higher thermal treatment temperatures increased the concentration of OVs, with BTO‐400 (BTO at thermal treatment temperature of 400°C) showing optimal ROS generation and piezocatalytic activity due to an appropriate concentration of OVs. However, BTO‐450 (BTO at thermal treatment temperature of 450°C) exhibited reduced ROS generation, as excess OVs can act as recombination centers for charges, reducing carrier mobility and ROS production [111]. Yang et al. explored the impact of OVs on the piezoelectric catalytic performance of ZnO crystals, finding that ZnO mesocrystals with moderate concentrations of OVs demonstrated superior piezoelectric catalytic activity. The presence of OVs increased electron delocalization and improved charge transfer, enhancing reactant capture. However, an excessive concentration of OVs diminished the piezoelectric response and suppressed overall performance [112].

### 5.3 Dopants

For piezoelectric materials, dopants can influence the crystal symmetry, carrier concentration, and electronic structure, which can ultimately impact their piezoelectric catalytic performance [113]. Regarding dopants, they significantly impact the piezoelectric catalytic performance by introducing defect energy levels that narrow the band gap, thus enhancing electron excitation opportunities [112]. Dopants like sulfur (S), lithium, lanthanum (La), cobalt (Co), and aluminum (Al) can also improve material conductivity, facilitate interfacial charge transfer [114], and induce local crystal distortions to enhance piezoelectric effects [115]. Lei et al. discovered that a sulfur‐doped BTO piezocatalyst (SDBTO) displayed optimal catalytic performance when the mass ratio of thiourea to BTO was 1:1. However, exceeding this ratio led to excessive oxygen vacancies which could detrimentally affect the catalytic capabilities, while insufficient doping failed to enhance catalysis [116].

### 5.4 Forming Composites

Besides modifying the intrinsic properties of piezoelectric materials, there is a growing body of literature exploring the combined application of piezoelectric materials with other materials to form composites, thus enhancing piezoelectric performance. For piezoelectric polymers, the incorporation of functional nanomaterials into the polymer matrix has been demonstrated to effectively enhance their piezoelectric performance characteristics. In contrast, inorganic piezoelectric nanoparticles exhibit significant performance improvement through the construction of heterojunction architectures with other materials.

#### 5.4.1 Incorporation of Functional Nanomaterials Into a Polymer Matrix

As mentioned in the previous section, PVDF is a typical piezoelectric polymer. Numerous studies have pointed that the addition of functional nanomaterials (such as TiO_2_, Fe‐rGO, BiVO_4_, PZT, BTO, NaNbO_3_, etc.) into its matrix can interrupt its symmetry structure and lead to the formation of a polar crystalline β‐phase, which consequently enhances the ferroelectric properties [41]. In addition, the inclusion of nanofillers in PVDF fosters electrostatic interactions with PVDF chains, influencing their orientation and boosting the piezoelectric response of the composites [117]. For instance, Li et al. enhanced piezoelectric performance of PVDF by embedding ZnO nanoparticles within PVDF fibers [118]. Liao et al. also developed a novel CBO/PVDF composite membrane by integrating the piezocatalyst Cu_3_B_2_O_6_ (CBO) with PVDF, which exhibited exceptional stability and piezoelectric performance [119]. Similarly, Shuai et al. filled BTO nanoparticles into PVDF matrix to form a composite material, which increased the dielectric constant of PVDF matrix [120].

#### 5.4.2 Forming a Heterojunction With Other Materials

To improve ROS production, the main strategy is to combine piezoelectric materials with other materials to form heterojunctions, where the polarized electrons excited by US at the interface can effectively migrate. Therefore, it can increase the yield of electrons while suppressing the recombination of electron–hole pairs [121]. For example, Wu et al. have deposited Au nanoparticles on the surface of piezoelectric BTO to form a metal/semiconductor Schottky structure [121]. However, the use of noble metals may lead to tissue toxicity, such as hepato‐renal injury [122]. Therefore, Feng et al. used bio‐compatible non‐noble metals to enhance the piezoelectric catalytic ability. They constructed heterogeneous interfaces of HNTM and MoS_2_, where the sonopiezoelectric polarization of MoS_2_ can improve the sonocatalytic ability of HNTM through augmenting the charge transfer of the heterogeneous interface [123]. In addition to metal modification, semiconductors have broad application prospects for forming heterojunctions with piezoelectric materials due to their excellent conductivity that facilitates electron transfer. Wang et al. synthesized MoS_2_ and Cu_2_O heterojunctions using a simple hydrothermal method. They modified MoS_2_ nanosheets with Cu_2_O as semiconductor and the enhanced SDT antibacterial ability through a sono‐piezoelectric polarization effect and mechanical force was observed [124].

## 6 Applications of Piezoelectric Biomaterials in Pathogenic Eradication and Tissue Regeneration

Piezoelectric biomaterials are being increasingly explored for their potential in medical applications, particularly in the areas of antibacterial treatment and tissue regeneration. These materials exhibit the distinctive property of generating electrical charges in response to mechanical stress, which can be harnessed to activate biological processes at the cellular level. This section details the specific types of materials used, the catalytic conditions under which they are effective, and the biological outcomes observed in these innovative applications.

### 6.1 Treatment for Antibacterial Therapy

Bacteria, as a type of microorganism, are almost everywhere in human life and are closely related to our life. Bacterial infection is a great threat and burden to human health [63, 125]. At present, the difficulty in treating bacterial infections is to kill the bacteria embedded in the biofilm. Traditional antimicrobial therapy usually involves the systemic use of antibiotics, but the excessive abuse of antibiotics can lead to the development of drug‐resistant bacteria, and this method cannot effectively deliver antibiotics to the infection sites with locally formed biofilms, resulting in limited clinical success [126]. Therefore, limiting the emergence of drug‐resistant bacteria and optimizing the delivery of antibiotics have become the focus of attention. As mentioned earlier, piezoelectric polarization can promote the micro electrolysis of water and catalyze the reaction on the cathode surface to produce ROS for sterilization [66]. According to the results of cell experiments, the amount of ROS produced is safe for normal mammalian cells [127].

Piezoelectric catalysts, through mechanical stress, can cause significant physical damage to the bacterial cell wall, leading to various detrimental effects on the cell [128]. This damage often manifests as cell lysis, loss of structural integrity, and eventual cell death. Specifically, the integrity of cell membranes is compromised, resulting in the leakage of vital cellular components and ions. Such breaches can cause a loss of membrane potential and disrupt crucial functions like nutrient absorption and osmotic balance, culminating in bacterial death [9]. Furthermore, this mechanical stress can disrupt biofilms, making bacteria more susceptible to antibiotics and immune system attacks. Additionally, the stress response in bacteria is activated, marked by the upregulation of stress‐related genes and proteins, which increases their vulnerability to external stressors. The piezoelectricity‐driven local electron discharge and resulting oxidative stress disrupt bacterial cell membranes and deplete their energy sources, effectively inhibiting bacterial activity [129]. This comprehensive mechanism illustrates how piezoelectric materials can effectively contribute to antibacterial strategies by exploiting mechanical forces to disrupt cellular processes and structures [62]. As a result, recent studies have increasingly focused on antibacterial strategies utilizing piezoelectric biomaterials, highlighting this approach as an innovative and promising strategy [66]. Table 2 below provides a summary of the use of piezoelectric biomaterials in combating microbial infections. This section will explain the antibacterial effect of piezoelectric biomaterials and their application in related bacterial infectious diseases.

**TABLE 2:** Representative ultrasonic‐activated piezoelectric biomaterials and their medical applications.

Application	Biomaterial	Piezoelectric phase	Morphology	Ultrasonic stimulation condition	Working site	Properties	Ref.
Soft tissue infection	SF‐MA/PEGDA/Ag@BT	BaTiO_3_	Nanoparticle	/	Skin	Accelerated the healing process of infected wounds	[130]
	PtRu/C_3_N_5_	C_3_N_5_	Nanosheet	/	Skin	Showed almost 100% antibacterial efficacy against four strains of bacteria	[131]
	ZnO@GDY	ZnO	Nanosheet	1.0 MHz, 1.0 W cm^−2^, 50% duty cycle	Skin	Exhibited significant antibacterial and antibiofilm activities	[132]
	BTO@MMSa	BaTiO_3_	Nanoparticle	1.0 MHz, 0.8 W cm^−2^, 50% duty cycle	Skin	Utilized macrophage membrane function for antibacterial purposes	[133]
	P(VDF‐TrFE)/ZnO/TPP	PVDF&ZnO	Composite film	/	Skin	Enhancing wound healing through photodynamic and piezocatalytic actions	[134]
	PVDF‐TrFE	PVDF	Nanofiber	40.0 kHz, 1.0 W cm^−2^	Cecal	Resisted endogenous bacterial infections	[72]
Osteomyelitis	RBC‐HNTM‐MoS_2_	MoS_2_	Nanosheet	1.0 MHz, 1.5 W cm^−2^	Marrow cavity	Eliminated the bone infection and suppressed inflammation and bone loss	[135]
	BTO/Ber	BaTiO_3_	Nanoparticle	1.0 MHz, 3.0 W cm^−2^, 50% duty cycle	Marrow cavity	Promoted anti‐inflammatory immune responses of macrophage and enhanced osteoblast differentiation	[136]
	BNT	BaTiO_3_	Nanotube	Ultrasonic wave and NIR irradiation	Marrow cavity	Improved bone reconstruction by enhancing osteoblast adhesion and differentiation	[137]
	g‐ZnN_4_‐MoS_2_	MoS_2_	Quantum dots	1.0 MHz, 1.5 W cm^−2^, 50% duty cycle	Marrow cavity	Suppressed the inflammation and bone loss	[138]
	BiFeO_3_/Ti_3_C_2_	BiFeO_3_	Nanoparticle	1.0 MHz, 1.5 W cm^−2^, 50% duty cycle	Marrow cavity	Treated deep infections effectively	[123]
	HN‐Ti_3_C_2_	Ti_3_C_2_	Nanosheet	1.0 MHz, 1.5 W cm^−2^, 50% duty cycle	Marrow cavity	Activated signaling pathways to promote osteogenic differentiation	[139]
	S‐TiO_2−x_/CeO_2_	S‐TiO_2_	Nanosheet	1.0 MHz, 1.5 W cm^−2^, 50% duty cycle	Marrow cavity	Generated electrical stimulation and activated osteogenic signaling pathways	[140]
Implant‐associated infections	PH‐CpBT	BaTiO_3_	Nanoparticle	1.0 MHz, 1. 0 W cm^−2^, 50% duty cycle	Femoral condyle bone	Promoted angiogenesis and osteogenesis in the body	[141]
	Al‐STNT	SrTiO_3_	Nanoparticle	1.0 MHz, 1.5 W cm^−2^, 50% duty cycle	Alveolar bone	Promoted bone integration after implantation	[142]
	BaTiO_3‐x_/LA	BaTiO_3_	Nanorod	1.0 MHz, 1.5 W cm^−2^, 50% duty cycle	Tibia	Promoted M2 polarization and enhanced osteogenic differentiation	[143]
	piezoTi	BaTiO_3_	Nanoparticle	1.0 MHz, 2.0 W cm^−2^	Subcutaneous tissue	Downregulated biofilm genes and activated PI3K‐Akt and MAPK pathways	[74]

#### 6.1.1 Treatment for Soft Tissue Infection

**FIGURE 4:**
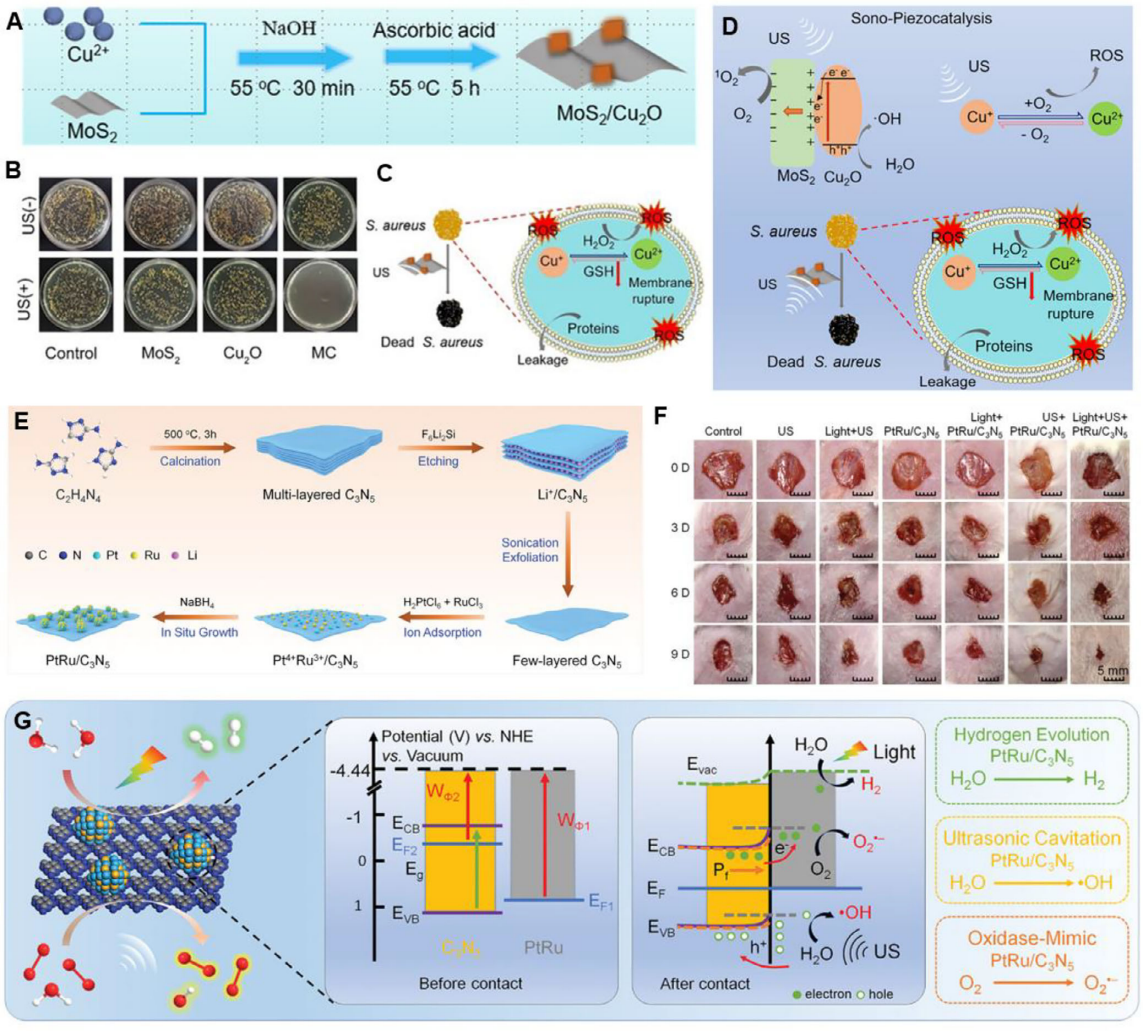
Antibacterial effect of piezoelectric biomaterials on planktonic bacteria. (A) Schematic illustration of the synthetic procedure of MC nanoparticle. (B) The spread plate images of *S. aureu*s of the different materials with and without US irradiation for 20 min. (C) The antibacterial mechanism diagram. (D) Sono‐piezocatalysis mechanism of antibacterial. Reproduced with permission [124]. Copyright 2023, The Liu et al. Wiley‐VCH GmbH (E) The synthetic route of PtRu/C_3_N_5_. (F) Representative images of wounds in different groups. (G) The possible catalytic mechanisms of PtRu/C_3_N_5_. Reproduced with permission. [131]. Copyright 2022, American Chemical Society.

Effective antibacterial activity for treating soft tissue infection can be achieved by optimizing the piezoelectric properties of biomaterials. Wang et al. engineered a heterojunction structure between MoS_2_ and Cu_2_O, termed MoS_2_/Cu_2_O (MC), which functions as a valence‐adjustable sonosensitizer [124]. This structure enhances ultrasonic catalytic effects by facilitating rapid electron transfer at the interface of the heterojunctions. Additionally, US regulation aids the conversion between Cu(I) and Cu(II), which effectively oxidizes glutathione (GSH) within bacteria, boosting antibacterial efficiency. After 20 min of US irradiation, MC demonstrates a 99.85% antibacterial rate against *S. aureus* and exhibits good biocompatibility, underscoring its potent bacterial killing capabilities (Figure 4A–D). Xu et al. selected PCL as the substrate and BTO NPs as piezoelectric catalysts to successfully construct piezoPCL [129]. According to their previous research, they also modified BTO NPs with Au cocatalysts, which can promote the faster production of reactive oxidizing substances under US irradiation [144]. After 5 min of low‐power US irradiation, the in vitro antibacterial rate of piezoPCL was 99.2% for *S. aureus*, and the in vivo antibacterial rate was 96.9%, demonstrating high anti‐reinfection ability at the same time. Similarly, Chen et al. constructed a piezoelectric catalytic hydrogel, SF‐MA/PEGDA/Ag@BT (SPAB), by depositing Ag NPs on the surface of BTO and encapsulating them in a hydrogel [130]. The results of in vitro antibacterial experiments indicated that SPAB had good piezoelectric catalytic performance and could effectively produce ROS after US irradiation. The antibacterial rates against *S. aureus* and *Escherichia coli* (*E. coli*) reached 99.96% and 99.23%, respectively. Masimukku et al. demonstrated that the antibacterial properties of single and few‐layers WS_2_ NFs also reached more than 99.99% to against the *E. coli* under US condition [145]. In addition to killing *S. aureus* and *E. coli*, piezoelectric materials also have a positive effect on MRSA and *P. aeruginosa*. Shi et al. grew an ultra‐small platinum‐ruthenium (PtRu) nanoalloy on the porous graphite carbon nitride (C_3_N_5_) nanosheets to form PtRu/C_3_N_5_ nanocomposite, and they hoped to use the piezoelectric field in the C_3_N_5_ nanozyme to couple the US energy conversion with the catalytic process of the nanozyme (Figure 4E–G) [131]. In addition, Dai et al. developed a flexible piezo‐phototronic composite film based on P(VDF‐TrFE)/ZnO/TPP, which synergistically integrates photodynamic and piezocatalytic therapies for enhanced wound healing [134]. Upon mechanical deformation and light exposure, the film generates ROS and a piezoelectric field that mutually amplify therapeutic effects. In vitro and in vivo results confirm accelerated wound repair, highlighting the potential of this strategy for non‐invasive treatment via daily physical activity and ambient light.

**FIGURE 5:**
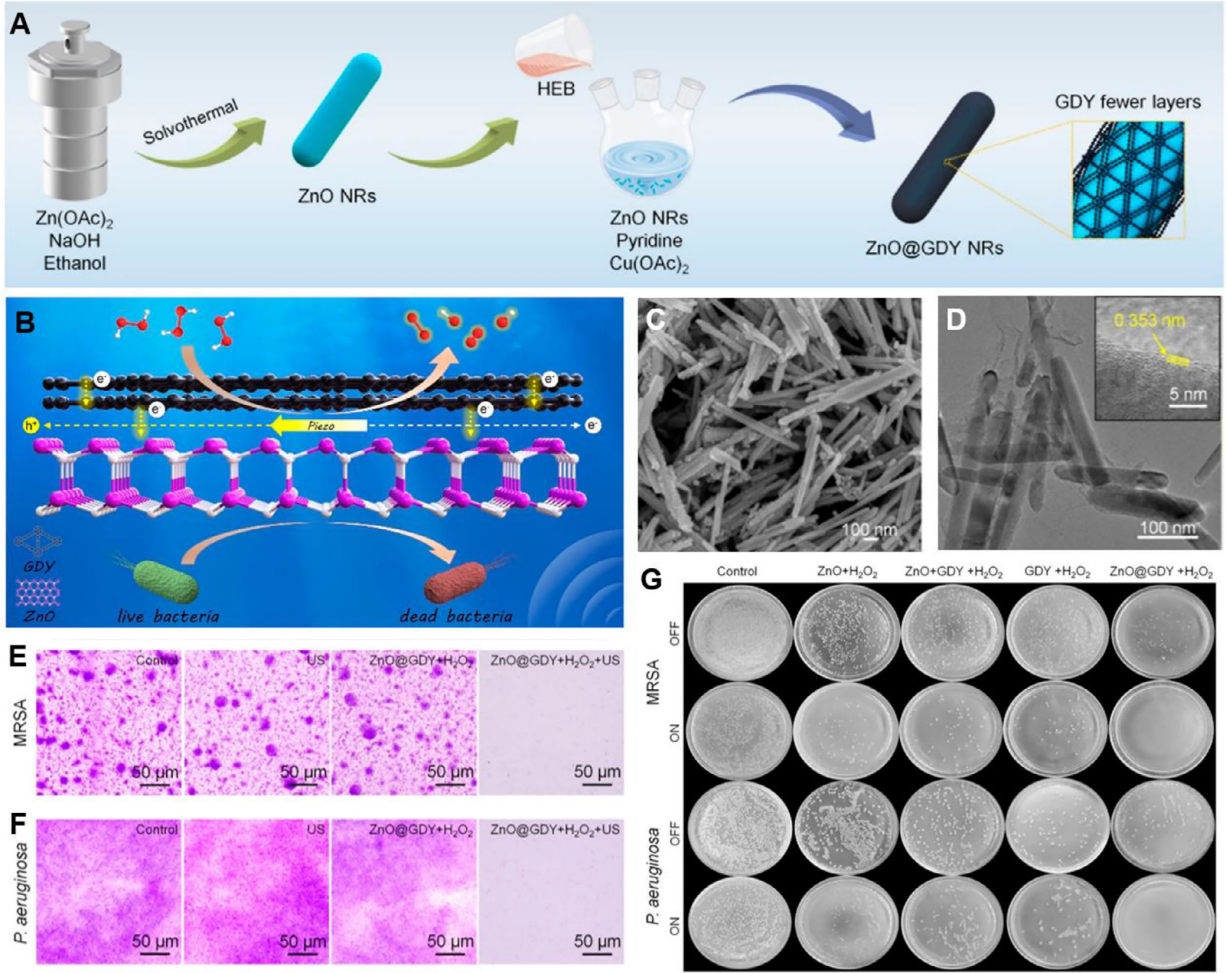
Antibacterial effect of piezoelectric biomaterials on bacterial biofilms. (A) Schematic illustration of the synthetic route of ZnO@GDY NRs. (B) Mechanism of antibacterial. (C) SEM image of ZnO@GDY NRs. (D) TEM image of ZnO@GDY NRs. (E, F) Images of crystal violet‐stained biofilm. (G) Digital photographs of colonies of MRSA and *P. aeruginosa* treated with ZnO@GDY NRs under US irradiation with the treatments of blank control, ZnO, GDY, and ZnO + GDY used as the control group. Reproduced with Permission [132]. Copyright 2022, Wiley‐VCH GmbH.

Piezoelectric biomaterials have demonstrated strong antibacterial efficacy not only against planktonic bacteria but also in the disruption of bacterial biofilms—one of the major challenges in current antimicrobial therapies. Zhao et al. pointed out that most existing antibacterial strategies overlook the critical role of EPS in biofilm resistance. As a protective matrix, EPS hinders the penetration of antibiotics and nanomaterials and can anchor to bacterial surfaces. Moreover, the electrochemical activity of the EPS layer suggests that modulating the local electrical microenvironment may destabilize its structure, thereby interfering with bacterial electron transport chains and inducing cell death. To exploit this mechanism, Zhao et al. combined a piezoelectric nanofiber film (PNF) composed of PVDF‐TrFE with US stimulation to disrupt the molecular integrity of EPS. In vivo studies using a rat cecal ligation and puncture (CLP) model demonstrated that piezoelectric films effectively damage bacterial biofilms by transferring surface charges into the biofilm matrix, thereby disrupting EPS structure and exerting notable antibacterial effects [72]. In another study, Bai et al. developed a heterostructured nanozyme composed of ZnO nanorods and graphdiyne nanosheets (ZnO@GDY NR), which exhibits exceptional piezocatalytic and peroxidase‐mimicking activities. Under ultrasound irradiation, this hybrid nanozyme catalyzes H_2_O_2_ decomposition and generates ROS, leading to potent antibacterial effects. In both in vitro and in vivo experiments, ZnO@GDY NR achieved nearly 100% antibacterial efficiency against multidrug‐resistant pathogens, including methicillin‐resistant *Staphylococcus aureus* (MRSA) and *Pseudomonas aeruginosa*. Quantitative crystal violet staining further confirmed the biofilm‐eradicating effect of the nanozyme. While extensive biofilm coverage was observed in the control, US‐only, and ZnO@GDY+H_2_O_2_ groups, only fragmented biofilm remnants remained in the group treated with ZnO@GDY+H_2_O_2_+US (Figure 5), over 80% of biofilm biomass was eliminated following this treatment [132]. These findings underscore the promise of hybrid piezoelectric nanozymes as effective strategies for biofilm disruption and the treatment of soft tissue infections.

As previously discussed, optimizing antibiotic delivery has become a central focus in antimicrobial therapy. Similarly, enhancing the delivery efficiency of piezoelectric antibacterial materials has garnered increasing attention. In recent years, various studies have explored the integration of hydrogels with piezoelectric components to develop innovative antibacterial dressings. However, Qian et al. identified several limitations associated with conventional antibacterial hydrogels, including irregular shapes, non‐uniform thickness, and complex fabrication procedures. To address these challenges, they proposed a piezocatalytic nanospray system as a flexible alternative to broad‐spectrum antibiotics. The key innovation of this study lies in the fabrication of macrophage membrane‐coated nanoparticles (MMSa), which possess specific bacterial recognition capabilities. These membranes were functionally integrated onto BTO nanoparticles to construct a targeted nanosystem termed BTO@MMSa. This system can be easily aerosolized into a nanospray using a nozzle‐equipped container, enabling convenient and localized application. Moreover, due to the presence of pathogen‐specific receptors on the macrophage membranes, BTO@MMSa exhibits preferential accumulation at infection sites while sparing normal tissues, thereby significantly reducing systemic toxicity and improving therapeutic precision [133].

In summary, piezoelectric biomaterials are expected to become one of the candidates to kill the planktonic bacteria or those embedded in the biofilm, providing sustained antibacterial effects and reducing potential adverse reactions for treating soft tissue infection.

#### 6.1.2 Treatment for Osteomyelitis

The treatment of osteomyelitis, a chronic inflammatory disorder, has become a major clinical challenge [146]. Clinical treatments for osteomyelitis involve the long‐term and high‐dose antibiotic administration, as well as multiple surgical debridement procedures [147]. However, the reasons for the limited success rate of treating osteomyelitis with antibiotics are as follows: (1) Long‐term systemic high‐dose use of antibiotics can lead to disruption of the immune response [148] and the emergence of drug‐resistant bacteria [149]. (2) Antibiotics generally do not treat the accompanying symptoms of osteomyelitis, including intense oxide conditions, immune response dysbiosis, and bone tissue loss. Thus, it is highly desirable to employ an antibiotic‐free, in situ, reparative and rapid strategy to treat drug‐resistant bacterium‐induced osteomyelitis. SDT, as a controllable, non‐invasive and highly tissue‐penetrating therapeutic method, offers a new opportunity for the treatment of deep tumors and MDR bacterial infections in patients with osteomyelitis [150]. Traditional sonosensitizers include TiO_2_ nanoparticles, porphyrins, noble‐metal nanoparticles, carbon‐based nanoparticles and piezoelectric nanoparticles (ZnO NPs, BTO NPs, etc.) [135]. When applying sonosensitizers to antibiotic therapy, there is often a dilemma of the scarcity of sonosensitizers [151] and insufficient catalytic efficiency. Therefore, identifying novel sonosensitizers and enhancing the catalytic efficiency of sonosensitizers are urgently needed. As mentioned before, piezoelectric materials are potentially efficient sonosensitizers because of their unique piezoelectric effect, the cavitation effect during US and the sonoluminescence from the collapsing bubbles.

However, literatures have pointed out that treating osteomyelitis directly with piezoelectric materials could pose a number of problems. Therefore, we need to modify the materials to achieve efficient treatment of osteomyelitis. For example, to address the challenge of treating bone infections with BTO or BTO‐based nanoparticles, which struggle under the harsh oxidative conditions of bone infections due to their poor antioxidant capabilities, Fu et al. developed a novel approach by encapsulating BTO with Berberine (Ber) to create BTO/Ber NPs [136]. Berberine is an isoquinoline alkaloid known for its potent ROS‐scavenging, anti‐inflammatory, and immunomodulatory properties, which include the regulation of dendritic cells and macrophages. This encapsulation significantly enhances the antibacterial activity of the nanoparticles, achieving a 99.80 ± 0.09% effectiveness against *S. aureus*. Further advancing the field of piezoelectric materials for infection treatment, Li et al. introduced a new composite, BiFeO_3_/Ti_3_C_2_, which combines Ti_3_C_2_ and BiFeO_3_. This material benefits from ferroelectric polarization and a Schottky junction that accelerates charge transfer at the interface [123]. Tested in vivo for treating osteomyelitis, BiFeO_3_/Ti_3_C_2_ demonstrated strong antibacterial performance under US treatment. After 2 days of US treatment, a significant reduction in *S. aureus* colonies was observed in bone marrow, liver, and kidney, with an antibacterial efficiency of 99.63% ± 0.16%. H&E staining of major organs like the lungs and spleen showed minimal inflammation, indicating effective treatment of osteomyelitis and mitigation of systemic damage caused by the bacterial infection. These findings underscore the potential of piezoelectric materials as effective sonosensitizers for targeted sonodynamic therapy of bacterial infections such as osteomyelitis.

**FIGURE 6:**
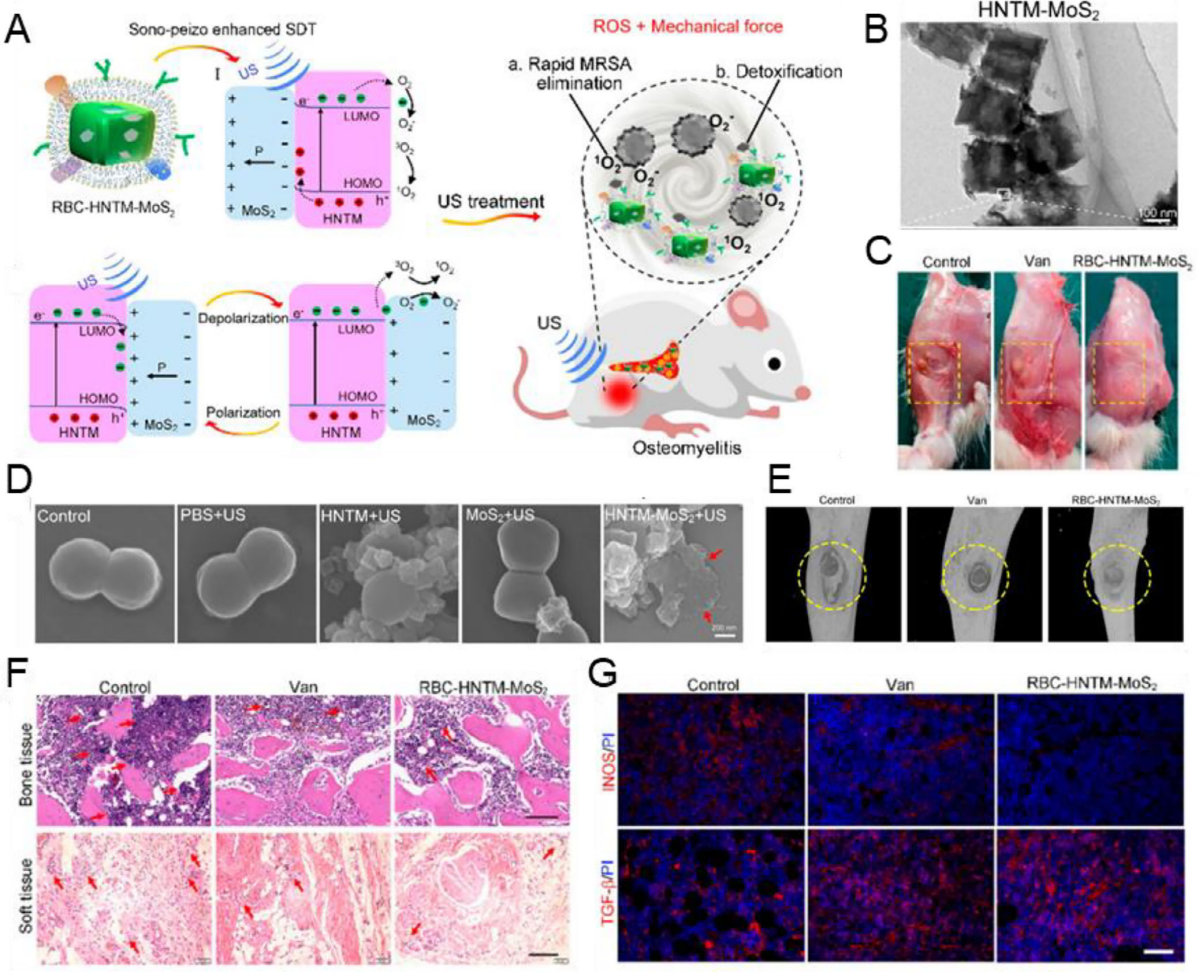
Application of piezoelectric biomaterials in the treatment of osteomyelitis. (A) Sonocatalytic mechanism of HNTM‐MoS_2_ and the efficient SDT treatment of osteomyelitis through rapid MRSA elimination and detoxification. (B) HRTEM images of HNTM‐MoS_2_. (C) Wound images of infected sites. (D) SEM images of MRSA treated by samples. (E) Micro‐CT results of the infected legs. (F) H&E staining of the infected bone and soft tissue. (G) Immunofluorescent staining of inducible nitric oxide synthase (iNOS; M1‐type) and transforming growth factor‐β (TGFβ; M2‐type) in the bone tissue. Reproduced with permission [135]. Copyright 2022, American Chemical Society.

To enhance the catalytic efficiency of sonosensitizers, noble metals such as Au and Pt can be used to enhance the sonocatalytic ability of sonosensitizers via supplying an extra electron transfer path [152], but they also lead to tissue toxicity such as hepato‐renal injury [122]. To address this problem, Feng et al. considered utilizing biocompatible non‐noble metals to augment the sonocatalytic ability. They combined hollow metal–organic framework (HNTM) with MoS_2_ nanosheets to form a bifunctional sonosensitizer with excellent sonodynamic antibacterial efficiency and osteogenic ability. This sonosensitizer can kill MRSA by generating ^1^O_2_ in combination with vortex effect under US stimulation, and the red blood cell membrane modified on the surface can also neutralize residual toxins in the bone marrow (Figure 6) [135]. Prior to this study, Feng et al. also developed a bifunctional sonosensitizer that consists of porphyrin‐like Zn single‐atom catalysts (g‐ZnN_4_) and MoS_2_ quantum dots to treat MRSA‐infected osteomyelitis. The experimental results show that it also has a good antibacterial effect on the treatment of MRSA‐infected osteomyelitis [138]. Similarly, Wang et al. also used HNTM and modified Ti_3_C_2_ nanosheets on its surface to form heterojunctions. Under US irradiation, the antibacterial rate of HN‐Ti_3_C_2_ against MRSA was 99.75%, and it generated acoustic currents that activated the calcium, Wnt, and TGF‐β signaling pathways, promoting osteogenic differentiation. In a rat model of tibial osteomyelitis infected with MRSA, HN‐Ti_3_C_2_ successfully eliminated the infection and significantly improved bone regeneration [139]. Wang et al. enhanced sonocatalytic therapy by doping S element into TiO_2_ and creating a heterojunction between TiO_2_ and CeO_2_ to accelerate the transfer of interface electrons. They successfully constructed S‐TiO_2−_ *_x_*/CeO_2_ sonosensitizer, which can kill 99.3% of *S. aureus* under US, while generating electrical signals and cerium ions which can activate the Wnt/β‐catenin signaling pathway, inducing human bone marrow mesenchymal stem cells (hBMSCs) to differentiate into osteoblasts. On day 7 of successfully constructing an in vivo osteomyelitis model, it was observed that the S‐TiO_2−_ *_x_*/CeO_2_ treatment group exhibited the lowest degree of inflammation and infection, as well as minimal bone destruction without any dead bone formation. These findings suggest that it can effectively eliminate osteomyelitis infection and promote bone regeneration [140].

#### 6.1.3 Treatment for Implant‐Associated Infections

At present, implants made of inert materials have been widely used in fields such as fracture fixation, spinal restoration, joint replacement, and dental treatment for missing teeth. However, in order for implants to achieve stable and good long‐term clinical efficacy, the threat of implant associated infection (IAI) cannot be ignored. At present, the main treatment measures for IAI include systemic use of antibiotics and mechanical removal of plaque, the former of which can lead to bacterial resistance issues. Based on this, Huang et al. proposed a novel and non‐invasive approach for treating IAI. They combined BTO with polydopamine and copper. And after US activation, piezoelectric carriers can be transferred to copper through polydopamine, achieving the valence state transition of copper ions. On one hand, Cu^+^ can catalyze H_2_O_2_ to promote the production of ·OH, and on the other hand, intracellular copper overload can promote bacterial copper apoptosis like death. The results of in vitro experiments showed that the polyetherketone ketone scaffold exhibited excellent antibacterial properties against both *S. aureus* and *E. coli* under US stimulation, providing ideas for non‐invasive treatment of IAI [141]. Xu et al. also proposed a non‐invasive approach. They believe that metal/piezoelectric nanostructures (BTO@Au) can be used on the surface of implants [129]. Under the activation of US, local ROS in the gap between the implant and tissue can be increased to combat infection. The antibacterial experiment results confirmed that this material is effective against various pathogenic bacteria that can cause implant infections, and its antibacterial performance did not significantly change after storage for about 12 months. They also proposed ideas for the future application scenarios of polymer implants with piezoelectric surfaces. Russo et al. did not combine piezoelectric materials on the surface of implants, but applied them to instruments for removing bacterial biofilms [153]. When periprosthetic joint infection (PJI) happens, pulse lavage (PL) debridement is the standard treatment used in debridement, antibiotics and implant retention (DAIR) for bacterial biofilm. However, the effect of PL in removing biofilm is not ideal, and the failure rate of DAIR is still high. To address this issue, they attempted to use a piezoelectric US scale (PUS) to remove bacterial biofilms from different orthopedic implant materials in vitro, and found that the efficiency of PUS was significantly improved compared to PL, indicating that the use of PUS can improve the success rate of DAIR.

**FIGURE 7:**
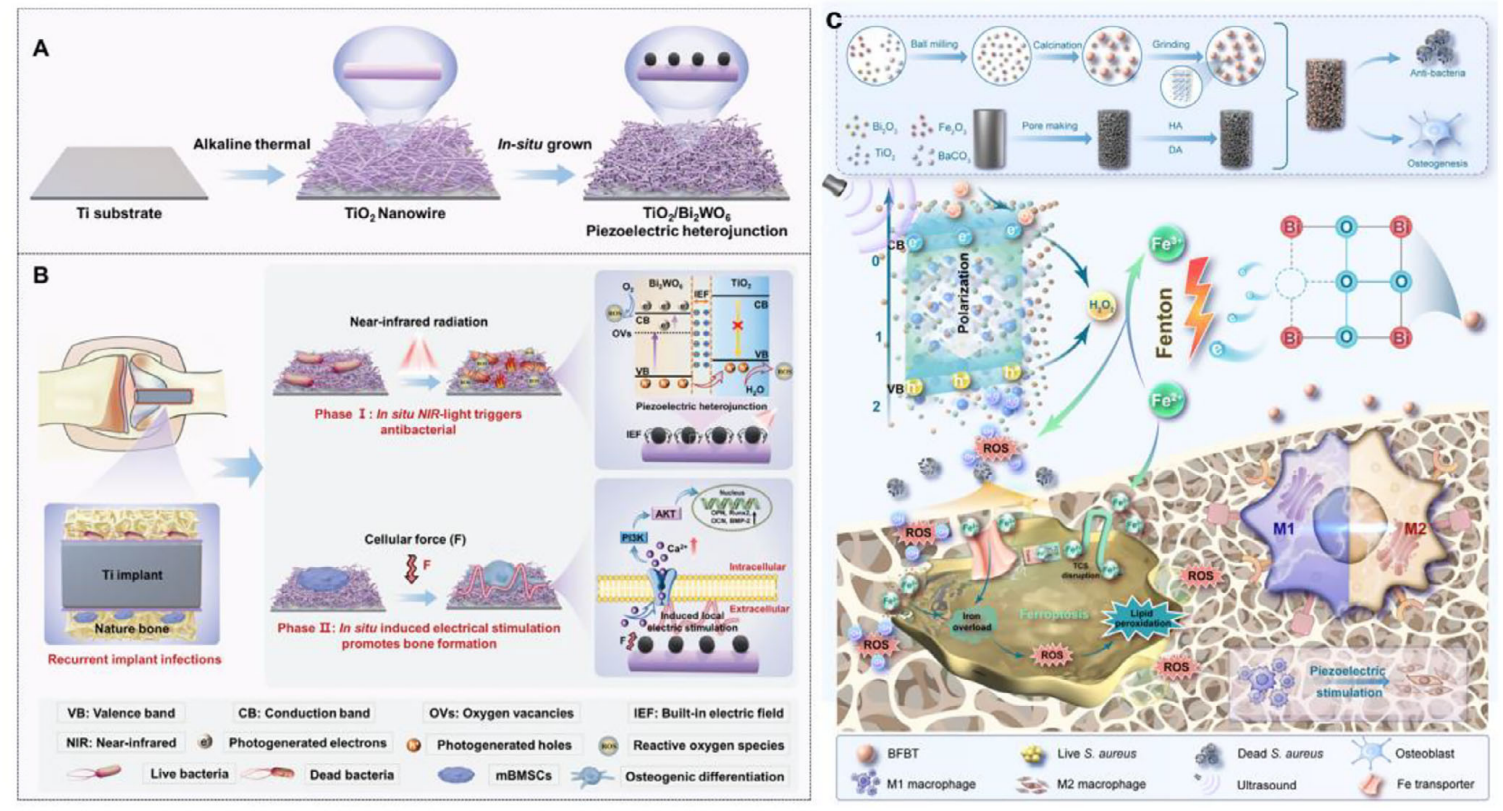
The piezoelectric biomaterials for promoting titanium implant infection treatment. (A) Constructing TiO_2_/Bi_2_WO_6_ heterojunctions directly on the surface of titanium implants, providing a structural basis for their functional properties. (B) Utilizing NIR light to trigger ROS and heat generation through both photodynamic and photothermal mechanisms, enabled by the built‐in electric field (IEF). Additionally, mechanical forces from cellular activity induce electrical stimulation that aids in bone formation. Reproduced with permission [154]. Copyright 2024, The Fan et al. Wiley‐VCH GmbH. (C) Sono‐piezoelectric tandem catalysis and bacterial iron metabolism disruption for implant infections [155]. Copyright 2025, Zheng et al., American Association for the Advancement of Science.

Heterojunctions, which involve the combination of two different semiconductors, exhibit unique electronic properties at their interfaces that facilitate various physical phenomena such as heat, light, and electricity generation. Piezoelectric materials, known for their ability to transform mechanical stress into bioelectric signals that aid in bodily self‐repair, face challenges related to bacterial adhesion on implant surfaces, potentially leading to complications like implant loosening and infections. Fan et al. have developed piezoelectric heterojunction arrays (TiO_2_/Bi_2_WO_6_) by integrating piezoelectric nanocrystals (Bi_2_WO_6_) onto TiO_2_ nanowires [154]. These heterojunctions not only demonstrate excellent biocompatibility but also leverage light‐cell force‐electric coupling to promote bone regeneration and eradicate pathogenic bacteria. Under near‐infrared light, these heterojunctions produce ROS and heat through photodynamic and photothermal therapy, respectively, effectively preventing postoperative infections. Additionally, the mechanical stress from stem cells growing on the implant activates these heterojunctions to generate electric fields that further facilitate bone integration (Figure 7A,B). Li et al. constructed metal piezoelectric heterostructures with antibacterial properties on titanium implants. Under US irradiation, piezoelectric Ti (piezoti) can downregulate biofilm genes. Macrophages stimulated by piezoelectricity also showed strong phagocytic and antibacterial activities by activating PI3K‐Akt and MAPK pathways [74]. Sun et al. developed a US‐responsive hybrid coating for titanium implants, engineered to combat MRSA infections [143]. This coating combines oxygen‐deficient BTO nanorod arrays with l‐arginine to leverage both piezoelectric and sonocatalytic properties under US. The design optimizes electron and hole separation while minimizing their recombination through oxygen defects, enhancing ROS production during US treatment. Additionally, the l‐arginine component releases NO which interacts with ·O_2_ to form peroxynitrite, intensifying oxidative attacks on bacterial cell membranes and DNA, thus disrupting their self‐repair mechanisms and hastening bacterial death. Moreover, NO also modulates macrophage polarization and enhances osteogenic differentiation, offering a dual therapeutic approach by combining sonodynamic therapy with immunomodulation for effective treatment of implant‐related infections. Zheng et al. developed a multifunctional Ba/Ti‐doped perovskite [(BiFe)_0.9_(BaTi)_0.1_O_3−_ *_x_*, BFBT] nanoreactor with oxygen vacancies as a piezoelectric sonosensitizer, integrating it into porous titanium implants with hydroxyapatite for US‐triggered antibacterial and osteogenic effects (Figure 7C) [155]. Under US conditions, BFBT facilitated piezocatalytic ROS and H_2_O_2_ generation, enabling enhanced CDT and ferroptosis‐like bacterial death via tandem catalysis and Fe‐mediated iron metabolic disruption. Above research representing a significant advancement in combating implant‐associated infections by piezoelectric biomaterials.

**FIGURE 8:**
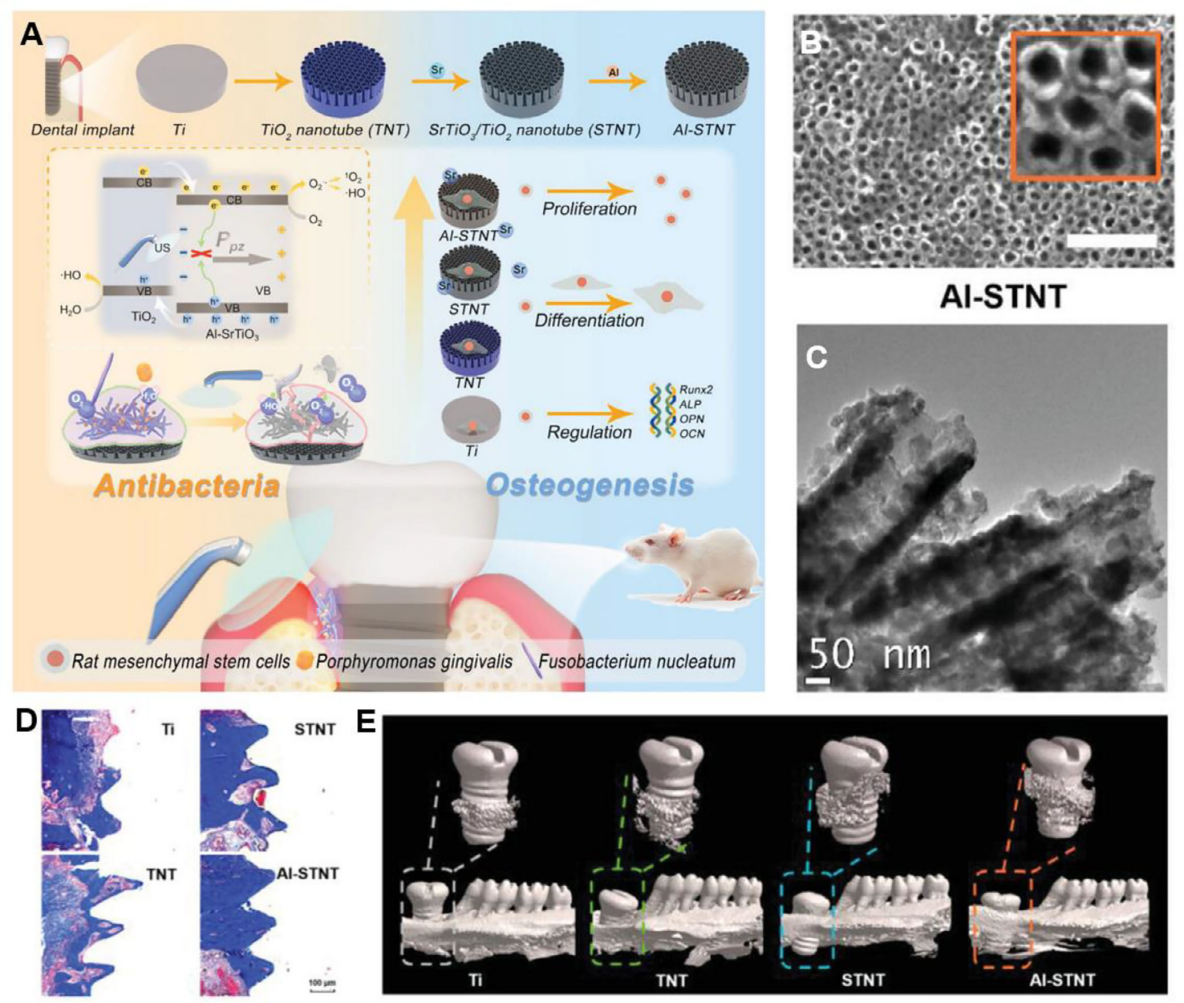
Application of piezoelectric biomaterials in the treatment of implant‐associated infections. (A) Schematic diagram of Al‐STNT synthesis with enhanced antibacterial sonodynamic therapy and inductive osteogenesis potential. (B) SEM images of Al‐STNT. (C) TEM image of Al‐STNT. (D) Masson staining of decalcified bone tissue slice. (E) Micro‐CT 3D reconstructions of the implants and surrounding bone tissues. Reproduced with permission [142]. Copyright 2024, Wiley‐VCH GmbH.

Dental titanium implants, widely used in the field of dentistry, are also a type of implant material and have gradually become the preferred treatment method for clinical restoration of dental defects [102]. Due to the direct contact between the implant and the alveolar bone, there is a lack of connective tissue attachment, which makes it easier for plaque to accumulate around the implant, leading to inflammation of soft tissues and damage to the alveolar bone. Recent studies have shown that the pathological environments of osteomyelitis and peri‐implantitis have certain similarities, and the use of US‐activated piezoelectric biomaterials can treat peri‐implantitis. Pan et al. believe that SrTiO_3_ crystals have good piezoelectricity, and Sr is also an essential trace element for the human body, which can activate multiple osteogenic signaling pathways. So, they successfully embedded Al ion doped strontium titanium/titanium dioxide nanotubes (Al‐SrTiO_3_/TiO_2_ nanotubes, Al‐STNT) onto the surface of titanium implants by doping Al, introducing oxygen vacancies, and forming heterojunctions with TiO_2_ (Figure 8). The in vitro experimental results showed that Al‐STNT can promote the proliferation, early adhesion, and spreading of osteoblasts, and upregulate the expression of osteogenic markers. In a rat in vivo model, the improved implant surface promoted stronger bone integration while effectively inhibiting the growth of bacteria around the implant [142]. Sun et al. point that the practical application effect of US responsive coatings is not good, and immunotherapy should be combined with sonodynamic therapy to enhance their antibacterial activity by stimulating immune cells. They believe that the NO^2−^ produced by US does not have strong antibacterial ability, so it is necessary for NO^2−^ to react with NO to generate peroxynitrite anion (ONOO^−^) with stronger antibacterial activity. So, they combined BTO with L‐arginine (LA), which can release NO, to form BaTiO_3−_ *_x_*/LA. In addition, NO can promote M1 polarization of macrophages. By implanting MRSA infected implants into the tibia of mice, an in vivo model of peri implantitis was established. The results showed that BaTiO_3−_ *_x_*/LA exhibited excellent antibacterial activity and improved osteogenic differentiation [143].

#### 6.1.4 Piezoelectric Degradation Bacterial Biofilm Fouling

**FIGURE 9:**
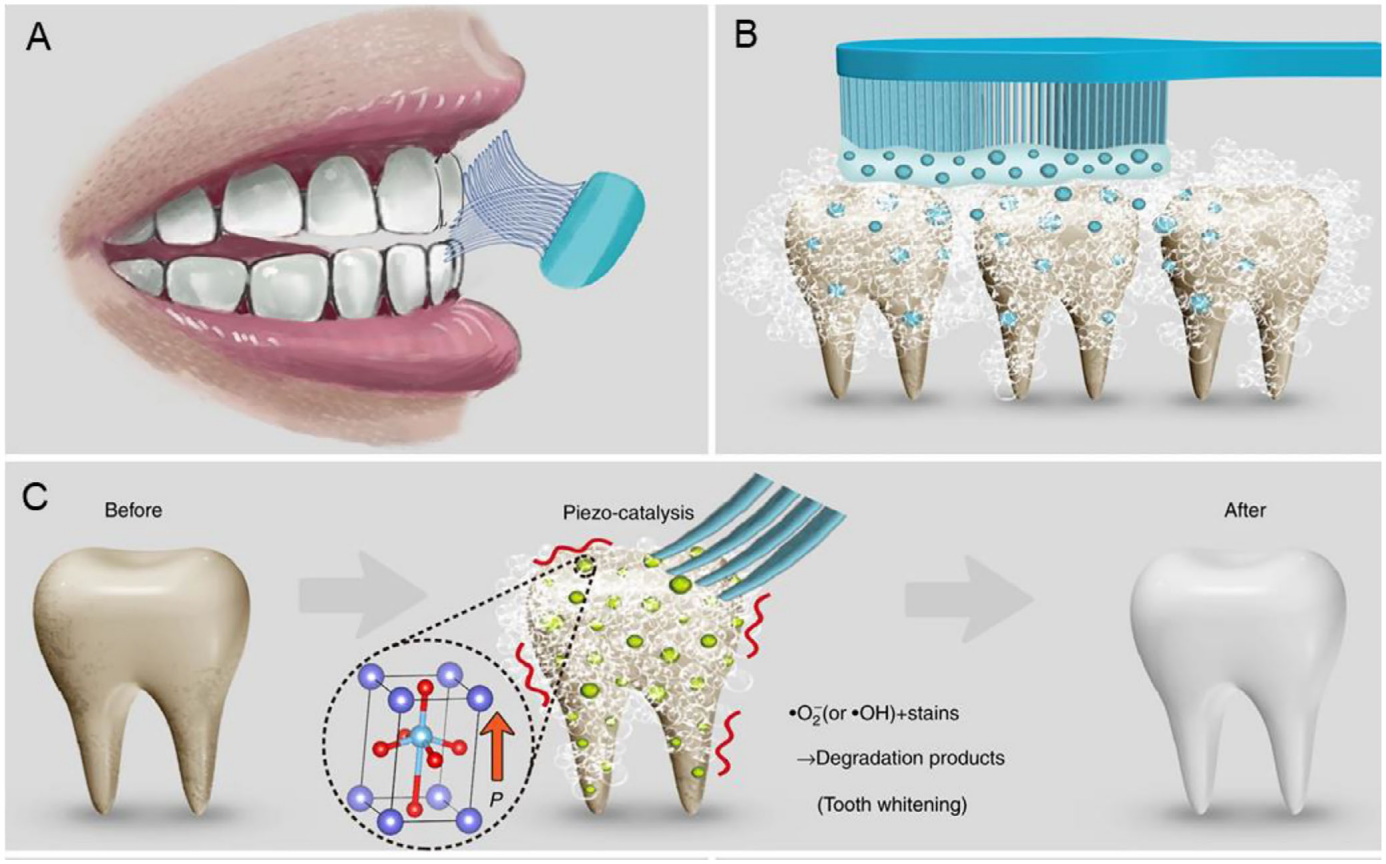
The piezo‐catalysis effect and its application in tooth whitening. (A) An illustration demonstrates the mechanical vibrations occurring between the toothbrush and teeth during routine toothbrushing. (B) Traditional tooth whitening methods utilize abrasive‐based toothpaste, where mechanical friction between the teeth and abrasive particles facilitates stain removal. (C) The innovative piezo‐catalysis effect‐based tooth whitening method replaces traditional abrasives with piezoelectric particles in the toothpaste. Reproduced with permission [33]. Copyright 2020, Wang et al. Springer Nature.

Fouling on tooth surface involves materials from the environment, such as macromolecules, microorganisms, and suspended particles, adhering to surfaces in a manner that can be either reversible or irreversible. This process can lead to significant health issues, including bacterial growth on tooth surface. In addition, a common everyday issue related to fouling is tooth staining, which affects the majority of people and has a negative impact on daily life. Typically, dentists use strong oxidants like H_2_O_2_ for teeth bleaching and employ professional equipment for polishing, but these methods can cause side effects such as tooth damage, gingival irritation, and acute pulpitis. An innovative approach reported by Wang et al. involves the use of piezoelectric BTO nanoparticles, synthesized hydrothermally. When used with an electric toothbrush that emits US vibrations, these nanoparticles generate ROS, specifically ·OH and ·O_2_, which effectively whiten teeth [33]. This method offers a nondestructive, harmless, and convenient alternative for teeth whitening, minimizing damage to tooth enamel and surrounding tissues (Figure 9). In addition, as the demand for aesthetic dentistry grows, there is a strong interest in developing at‐home tooth whitening solutions that are both effective and biologically safe. Piezocatalysis has emerged as a promising alternative to traditional peroxide bleaching, which can harm tooth enamel and gums. Recent advancements by Deng et al. have introduced biocompatible and biodegradable polylactide particles [156]. These particles are constructed from interlocking crystalline lamellae, designed hierarchically to enhance piezocatalytic activity and biosafety significantly. By fine‐tuning the chain conformation within the lamellae and managing the porosity of the lamellae network at both nano‐ and microscales, these particles achieve exceptional piezoelectric properties through improved dipole alignment and mechanical deformability. This unique structure increases the surface area, enhancing the piezocatalytic effect that can be activated simply by toothbrushing. This novel approach efficiently removes stains from substances like black tea and coffee without damaging the tooth enamel.

### 6.2 Treatment for Tissue Regeneration

Tissue engineering is a transformative approach that employs tissue regeneration techniques to replace or repair damaged organs and tissues, particularly useful in cases where traditional therapies fail. This method is vital for restoring body parts impaired by congenital defects, trauma, or diseases such as cancer. One of the key innovations in this field is the use of scaffolds made from piezoelectric materials, which offer distinct advantages due to their ability to provide electrical stimulation to cells. This stimulation triggers various phenotypic and genetic changes in the cells, significantly speeding up the tissue regeneration process. By harnessing the electrical properties of piezoelectric materials, these scaffolds actively promote the healing and functional restoration of affected tissues [157]. Bioelectricity plays a crucial role in regulating cellular and tissue functions via electrical stimulation. It is naturally generated in tissues and organs that respond to mechanical stimuli, as well as in biomolecular structures with inherent piezoelectric properties, a process known as the piezoelectric effect. Motivated by these natural bioelectric phenomena, researchers are actively developing synthetic piezoelectric biomaterials designed to enhance tissue regenerative capabilities by mimicking these biological processes [158]. These advanced materials aim to harness and replicate the body's innate ability to generate and respond to electrical signals, thereby promoting healing and regeneration. Due to its ability to generate charges or potentials under mechanical deformation, piezoelectric materials have shown significant application prospects in promoting bone regeneration, accelerating wound recovery, and supporting nerve repair. Table 3 below provides a summary of the use of piezoelectric biomaterials in enhancing tissue regeneration.

**TABLE 3:** Representative piezoelectric biomaterials in tissue regeneration.

Application	Biomaterial	Piezoelectric phase	Morphology	US stimulation condition	Working site	Properties	Ref.
Skin regeneration	Au@BTO	BaTiO_3_	Nanoparticle	1.0 MHz, 1.5 W cm^−2^	Skin	Promoted fibroblast migration	[159]
	BTO@ZIF‐8/CIP	BaTiO_3_	Nanoparticle	1.0 MHz, 1.5 W cm^−2^, 50% duty cycle	Skin	Promoted cell migration and wound healing	[160]
	CMCS‐TA/FWO_2_ gel	FeWO_4_	Nanorod	1.0 MHz, 1. 0 W cm^−2^, 50% duty cycle	Skin	Promoted skin regeneration, inhibited inflammatory response, increased collagen deposition and promoted angiogenesis	[161]
	PGCC film	PLLA	Nanofiber	1.0 MHz, 1.2 W cm^−2^	Skin	Accelerated healing of normal as well as infected wounds	[162]
	BTO‐400	BaTiO_3_	Nanoparticle	40.0 kHz, 1.5 W cm^−2^	Skin	Induced a rapid and efficient sterilization as well as skin tissue repair	[163]
	OHA/THM‐APMH	BaTiO_3_	Nanoparticle	1.0 MHz, 1.5 W cm^−2^	Skin	Effectively accelerated the healing of infected cutaneous incisions	[164]
	PLLA	PLLA	Nanofiber	40.0 kHz, 0.326 W cm^−2^	Skin	Enhanced the expression of typical genes during wound healing process	[165]
Bone regeneration	Se@BTO	BaTiO_3_	Nanoparticle	1.0 MHz, 1.5 W cm^−2^, 50% duty cycle	Condyle defect	Promoted the osteogenic differentiation	[166]
	SDBTO‐1	BaTiO_3_	Nanoparticle	1.0 MHz, 1.5 W cm^−2^, 50% duty cycle	Tibial defect	Depressed inflammation and improved bone regeneration	[116]
	PHB@ZnO NFs	PHB, ZnO	Nanofiber (PHB), Nanoparticle (ZnO)	1.0 MHz, 0.5 W cm^−2^	Shin‐bone defect	Facilitated adhesion, migration and recruitment of stem cells	[167]
	PDA@PVFT/GO	P(VDF‐TrFE)	Membrane	100 kHz, 0.7 W cm^−2^	Calvarial bone defect	Enhanced osteogenic differentiation and M2 polarization of BMSCs	[168]
	TC@BTO	Ti_3_C_2_、BaTiO_3_	Nanoparticle (BTO), Nanosheet (Ti_3_C_2_)	1.07 MHz, 0.309 W cm^−2^,	Cranial defect	Activated Wnt signaling pathway to promote bone regeneration	[169]
	piezoPCL	BaTiO_3_	Nanoparticle	US wave and NIR irradiation	Alveolar bone defect	Reduced periodontal inflammation and increased bone tissue regeneration	[80]
	GelMA + t‐BTO	BaTiO_3_	Nanoparticle	1.0 MHz, 0.6 W cm^−2^, 50% duty cycle	Alveolar bone defect	Promoted ATP synthesis and initiated PDLSC osteogenic differentiation	[170]
Cartilage healing	dECM+ Gel‐PC	Diphenylalanine	Hydrogel Scaffold	/	Cartilage defect	Attract bone marrow MSCs to migrate and differentiate into chondrocytes	[171]
	Piezo@CR	BaTiO_3_	Microspheres	1.0 MHz, 0.5 W cm^−2^	Cartilage defect	Promoted regeneration similar to surrounding normal cartilage tissue	[172]
Nerve regeneration	PCL/PVDF	PVDF	Nanobeam	1.0 MHz, 0.5 W cm^−2^	Sciatic nerve	Promoted pro‐regeneration Schwann cell functions, neuronal growth and axonal maturity	[173]
	PLA/KNN@PDA	PLA	Nanofiber	1.0 MHz	Spinal cord	Promoted neural stem cell differentiation and endogenous angiogenesis	[174]
	ND‐SENS	PLLA	Membrane	1.0 MHz, 1.0 W cm^−2^	Sciatic nerve	Promoted axon growth and repair sciatic nerve well	[175]

#### 6.2.1 Skin Regeneration

**FIGURE 10:**
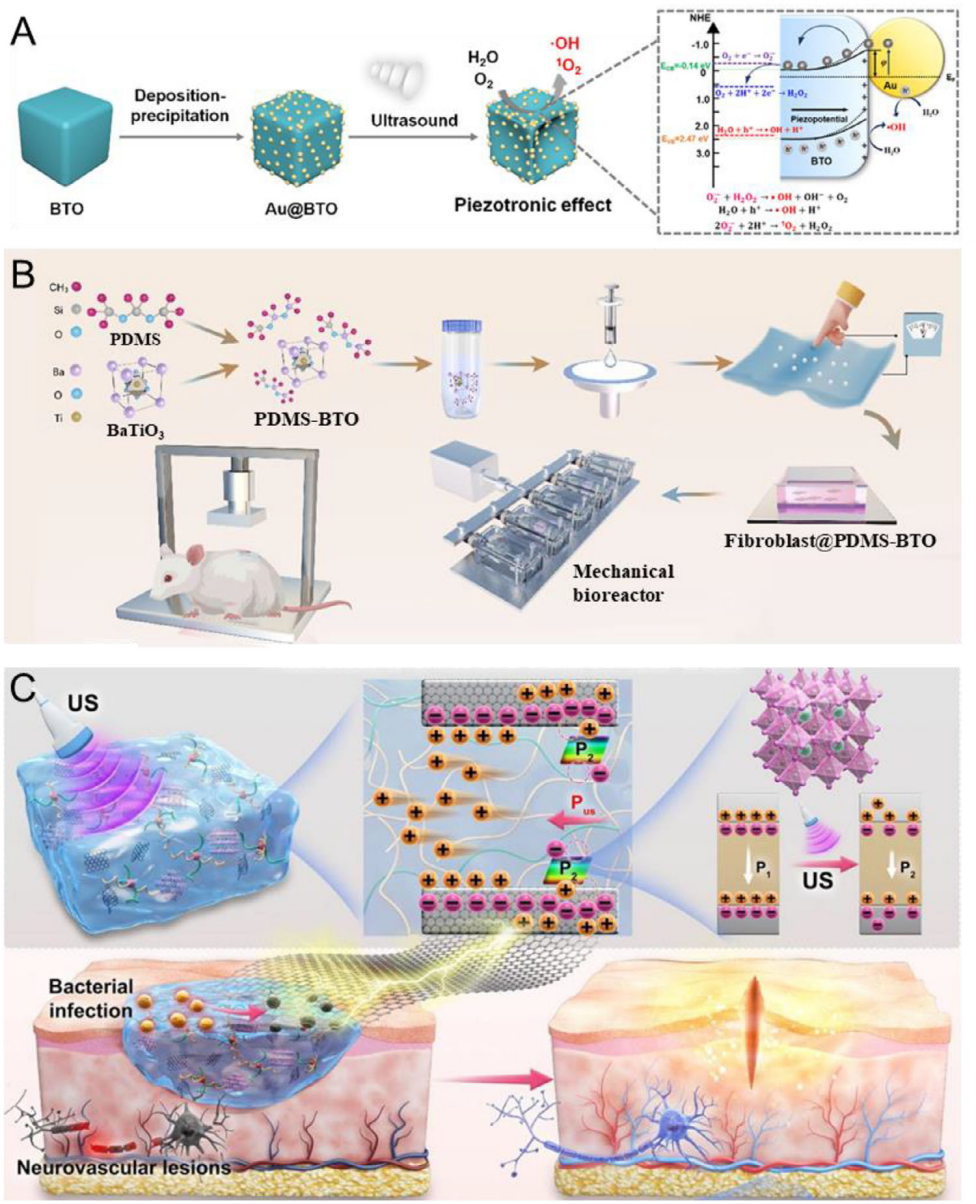
Application of piezoelectric biomaterials in skin regeneration. (A) Schematic illustration of piezoelectric mechanism for Au@BTO. Reproduced with permission [159]. Copyright 2021, Elsevier Inc. (B) A self‐powered repetitive mechanical impacts‐electrical stimulation (RMI‐ES) system, based on BaTiO_3_/PDMS piezoelectric composite films. Reproduced with permission [177]. Copyright 2024, Tsinghua University Press. (C) Diagram depicting the US‐triggered electromechanical transduction process mediated by hybrid hydrogel and its therapeutic effect in diabetic skin regeneration. Reproduced with permission [178]. Copyright 2025, Elsevier Ltd.

The skin, serving as the primary protective barrier between the human body and external elements, plays a vital role in preventing microbial invasion. Therefore, developing protective strategies that enhance the skin's natural barrier functions while effectively combating pathogens is critical. Several skin damage is severe and is likely to lead to the formation of chronic wounds due to the disruption of its integrity and the interference with biological functions. Conventional approaches to chronic wound management encompass localized debridement, therapeutic dressing application, systemic antibiotic administration, and biophysical stimulation therapies [176]. However, these established protocols often prove inadequate in sustaining the precisely regulated microenvironment for skin regeneration and delivering targeted bioactive stimulation required. In response to this issue, He et al. enhanced the ROS generation ability of BTO by introducing Vo, and confirmed that the Vo concentration in BTO was the most suitable when the calcination temperature was 400°C. BTO‐400 has excellent ability to generate ROS which can promote fibroblast migration for tissue repair by initiating cascade redox signals. Notably, the in vivo results indicate that BTO‐400 can accelerate skin healing through SDT therapy. Of all the bacterial infection groups, the BTO‐400 + US group had the fastest wound healing effect [163]. As was previously noted, doping metal ions to improve piezoelectric performance is another strategy in addition to introducing oxygen defects into piezoelectric materials. Wu et al. created the piezoelectric nanocomposite material Au@BTO, which allows BTO to be modified with Au nanoparticles to form a Schottky junction. US can trigger the piezoelectric effect of Au@BTO, thus promoting the separation and migration of charge carriers at the piezoelectric/metal interface to effectively increase the production of ROS. The treatment of Au@BTO + US significantly improved the wound healing rate of mice. From the histological analysis, mice showed intact epidermal dermal connection and the appearance of new hair follicles, indicating that the wound healing process was relatively fast. It was proved by scratch experiment that Au@BTO can also promote the migration of fibroblasts and macrophages for tissue repair (Figure 10A) [159].

The interdisciplinary integration of medicine and engineering has driven the development of innovative therapeutic delivery systems, particularly through bioengineered formulations such as adhesive transdermal patches and tunable hydrogel matrices, which demonstrate enhanced biocompatibility and site‐specific therapeutic efficacy in accelerating skin regeneration. For example, Xu et al. developed a BaTiO_3_/polydimethylsiloxane (PDMS) piezoelectric composite film that integrates the biocompatibility and flexible wearability of PDMS with the piezoelectric properties of BTO (Figure 10B). This composite film effectively harvests mechanical energy from rats' natural bodily movements and converts it into electrical stimulation, which accelerates the cell cycle, promotes fibroblast proliferation, and significantly enhances wound healing [177]. Hydrogel dressings have attracted widespread attention in wound management due to their stretchable and flexible nature, which enables effective wound coverage while maintaining an optimal moist environment to facilitate wound closure. Wang et al. engineered a gelatin/polyvinyl alcohol (PVA) interpenetrating polymer network (IPN) composite hydrogel, doped with piezoelectric potassium sodium niobate (KNN) nanocrystals and reduced graphene oxide (rGO) (Figure 10C). This multifunctional hydrogel exhibits exceptional self‐healing capability, skin‐adhesive properties, and electrical conductivity. Remarkably, it not only supports general wound healing but also promotes neurovascular regeneration and suppresses bacterial infections, thereby significantly accelerating the healing process of diabetic wounds [178].

**FIGURE 11:**
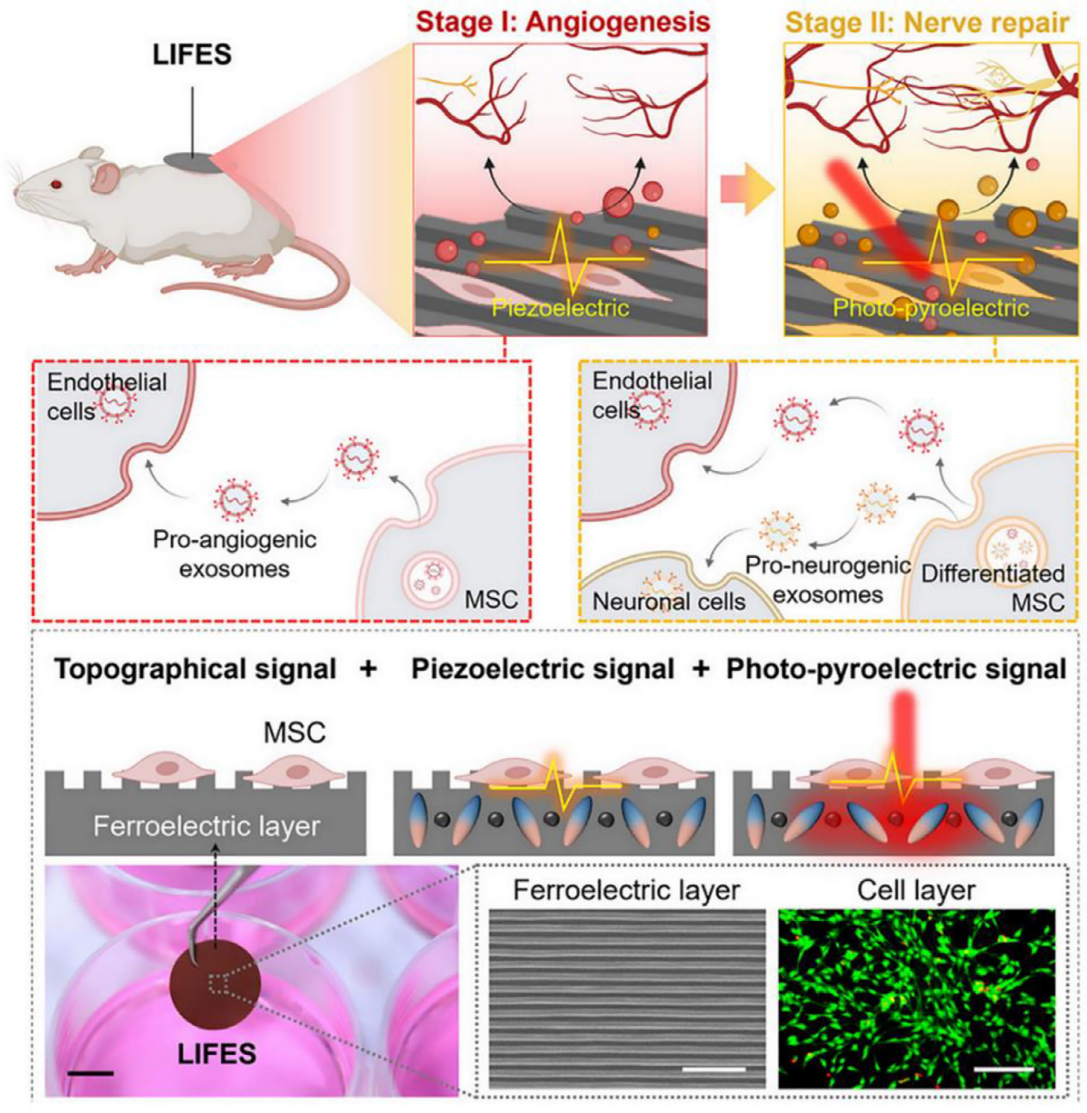
A ferroelectric living interface for fine‐tuned exosome secretion toward physiology‐mimetic neurovascular remodeling. Reproduced with Permission [179], Copyright 2024, Elsevier Inc.

Moreover, regenerative medicine and tissue engineering strive to restore the normal function of tissues or organs by establishing vascular neural networks, which are crucial for their success. Traditional methods often fall short as they do not adequately replicate the natural, sequential physiological processes that differentially target various cell types across different stages, thus impeding effective neurovascular remodeling. Peng et al. developed a novel approach with a ferroelectric living interface (LIFES), which fine‐tunes exosome secretion through a combination of topographic and electrical signals—both piezoelectric and photoelectric [179]. This interface includes a ferroelectric layer made from polydopamine and poly(vinylidene fluoride‐*co*‐trifluoroethylene) with nanogrooves, coupled with a living cell layer of mesenchymal stem cells (Figure 11). This innovative structure allows for the continuous production of bioactive exosomes, facilitating multi‐targeted and phase‐specific paracrine regulation that closely mimics natural physiological processes for neurovascular remodeling. Remarkably, LIFES has shown effective results in promoting neurovascular remodeling, even in challenging diabetic wound models.

#### 6.2.2 Bone Regeneration

Because bone regeneration is a complicated process, it continues to be a major concern in multiple fields of medicine. Traditional strategies including the use of autografts or allografts, are utilized for treating bone fractures. However, current research has indicated that the above approaches have limitations. Thus, the application of synthetic bone substitutes has been considered during the past few decades to aid in bone regeneration. Among various strategies, piezoelectric stimulation is a newly emerging field that aims to promote the formation of new bone tissue. In this regard, piezoelectric composite fiber scaffolds with excellent antibacterial properties can also be used for small molecule drug delivery, and piezoelectric polymers have shown significant antibacterial effects in different tissue engineering applications in SDT therapy [180]. Polyhydroxybutyrate (PHB) is a polymer scaffold and has drawn great attention for its biocompatibility, biodegradability and piezoelectric nature. The successful application of PHB has been reported in various biomedical fields, such as wound healing and regeneration of nerves and bone tissue [181]. Despite the above advantages, the sufficient mechanical properties and lower piezoelectric coefficient of PHB polymers limit their further clinical application in providing a bone‐forming microenvironment [182]. Chen et al. proposed a kind of scaffold based on piezoelectric PHB, which was loaded with chitosan (CS) and subsequently coated with ZnO to improve the mechanical strength and capability of generating local electric fields. Compared to those of the CS aerogels, the ECM‐like structure of the nanofiber scaffolds increased osteogenesis and the adhesion and migration of the BMSCs. The US‐driven electrical stimulation based on the piezoelectric nanofiber‐aerogels accelerated the differentiation of osteoblasts and promoted bone formation by activating the calmodulin/calcineurin/nuclear factor of activated T‐Cells (CaM/CaN/NFAT) signaling pathway [167].

In recent years, some scholars have found that piezoelectric materials can regenerate bone under the condition of non‐bacterial infectious diseases. Diabetes is a metabolic disease characterized by systemic hyperglycemia and inflammation [183]. Compared with normal people, the bone healing process of diabetes patients is usually slow and difficult, which is mainly due to the accumulation of ROS caused by oxidative stress in the microenvironment. Excessive ROS can affect intercellular signaling and is detrimental to bone regeneration. Therefore, Sun et al. prepared a piezoelectric catalytic nanocomposite membrane composed of polarized graphene oxide/ poly(vinylidene fluoridetrifluoroethylene) (GO/P(VDF‐TrFE)) ferroelectric membrane and polydopamine (PDA). The innovation of this study lies in, firstly, PDA@GO /P (VDF TrFE) can achieve antioxidant cycle and effectively alleviate oxidative stress in the process of diabetes tissue repair under US. Secondly, it can induce M1 macrophages to transform into M2 phenotype. Thirdly, under hyperglycemic conditions, it can more easily reactivate BMSCs and significantly upregulate the expression of osteogenic related genes (Runx2, Ocn, Opn) [168]. These results indicate that under the condition of diabetes, piezoelectric catalytic therapy is beneficial to immune regulation and bone formation, and has great potential application value in the field of bone regeneration. Many studies have shown that piezoelectric biomaterials have anti‐tumor effects, therefore they also have good regenerative effects on bone defects caused by tumors. Lv et al. constructed a heterojunction between BTO and atomic tin Ti_3_C_2_ (TC) to form TC@BTO and studied its therapeutic effect on bone defects caused by osteosarcoma (OS) surgery. Under continuous irradiation of US and near‐infrared, TC@BTO exhibited excellent photothermal conversion and ROS generation characteristics, which could lead to ferroptosis of tumor cells. In addition, the generated electrostatic stimulation can promote osteogenic differentiation of BMSCs. RNA sequencing results indicated TC@BTO promotes bone regeneration by activating the Wnt signaling pathway [169]. In summary, this study provides a novel strategy for piezoelectric biomaterials to induce bone regeneration in the tumor microenvironment.

**FIGURE 12:**
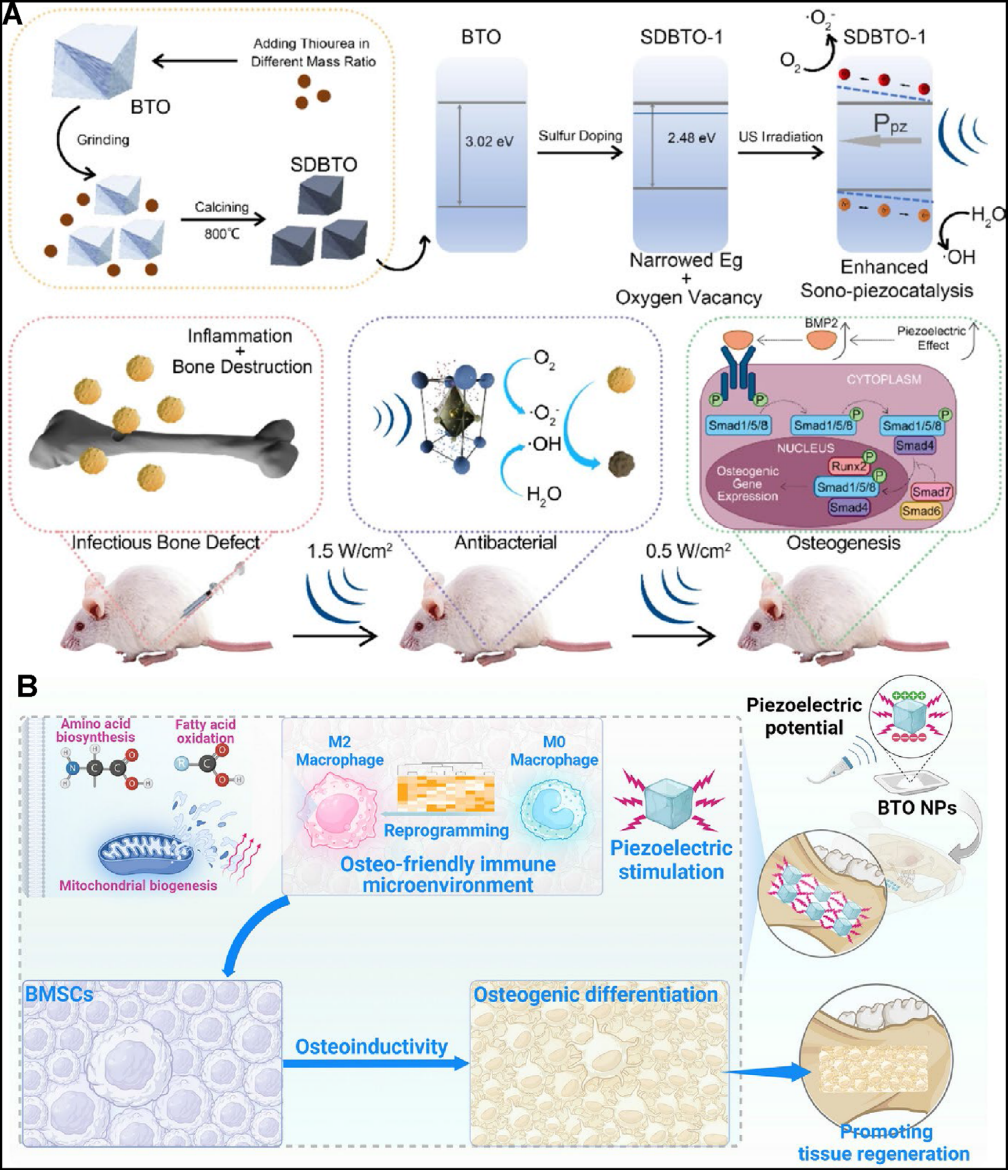
Application of piezoelectric biomaterials in bone regeneration. (A) Piezocatalysis mechanism of antibacterial and osteogenic therapy by SDBTO‐1. Reproduced with permission [116]. Copyright 2022, Elsevier. (B) Piezoelectric stimulation promotes osteogenic differentiation of BMSCs and alveolar bone tissue regeneration via macrophage reprogramming. Reproduced with permission [188] Copyright 2024, The Sui et al. Exploration published by Henan University and John Wiley & Sons Australia, Ltd.

During the disease process of bone defects, it is difficult to ignore the possibility of bacterial infection. Piezoelectric biomaterials also have applications for treating bacterium‐infected bone defects. Lei et al. constructed a US‐responsive SDBTO through sulfur doping and demonstrated that when the mass ratio of thiourea to BTO is 1:1, SDBTO has the best piezoelectric catalytic performance(Figure 12A). The antibacterial rate of SDBTO‐1 against *S. aureus* was 97.12%. At the same time, under mild medical US activation, SDBTO‐1 generates a medium to equal piezoelectric signal in vitro by upregulating TGF‐β signaling pathway, thus promoting osteogenic differentiation of human bone marrow mesenchymal stem cells. In vivo experiments have also confirmed that SDBTO‐1 can successfully treat tibial defects in rats infected with *S. aureus*, inhibit inflammation, and significantly improve bone regeneration [116]. Similarly, Lei et al. fabricated a US‐responsive selenium modified BTO nanoparticle (Se@BTO NP) that exhibited significant antibacterial and bone regeneration effects [166]. Periodontitis is a destructive disease that impacts oral health, and the bacteria and their metabolites in the plaque are its initiating factors. Periodontitis usually leads to the absorption of alveolar bone, resulting in premature tooth loss [184, 185]. Therefore, controlling the formation of plaque and guiding bone regeneration are key to the treatment of periodontitis. The commonly used clinical treatment methods include periodontal initial therapy, drug therapy, surgical treatment, etc. Statistics show that the clinical success rate of non‐surgical treatment is relatively low, only 39% [186, 187], so it is urgent to find a better non‐surgical treatment method. Roldan et al. believe that a piezoelectric hydrogel (piezoGEL) can be made by using the injectable and biocompatible properties of hydrogels and the piezoelectric properties of BTO, which may be injected into periodontal pockets to remove plaque. In vitro experiments have confirmed that piezoGEL has a certain antibacterial effect on *P. gingivalis* which is a crucial pathogen involved in the development of periodontitis. In addition, piezoGEL significantly enhances the vitality and osteogenic differentiation of BMSCs by upregulating RUNX2, COL1A1, and ALP. They constructed a periodontitis mouse model and found that PiezoGEL effectively reduced periodontal inflammation and increased bone tissue regeneration [80]. Recently, Liu et al. also prepared piezoelectric hydrogels containing BTO NPs, and studied the osteogenesis in periodontitis. It was found that this hydrogel could first initiate the osteogenic differentiation of periodontal ligament stem cells (PDLSC) by regulating energy metabolism. Secondly, it can induce macrophages to polarize towards M2, rebuilding the local regenerative microenvironment. Finally, piezoelectric stimulation under US conditions also promoted in situ regeneration of periodontitis induced in vivo defects in rats [170]. Immunomodulation is increasingly recognized as a valuable approach for enhancing bone regeneration. Yet, the development of a bone immunomodulatory biomaterial that effectively responds to the mechanical stresses unique to the alveolar bone under occlusal stress remains a significant challenge. Addressing this, Sui et al. pioneered a wireless piezoelectric stimulation system using piezoelectric hydrogels doped with BTO NPs [188]. This system harnesses piezoelectric potential to modulate macrophage activity, specifically reprogramming macrophages to the M2 phenotype, which in turn fosters osteogenic differentiation in BMSCs (Figure 12B). RNA sequencing analyses revealed that this macrophage M2 polarization is intricately linked to metabolic changes, including enhanced amino acid biosynthesis and fatty acid oxidation. Demonstrating practical efficacy, this innovative hydrogel has shown promising results in promoting endogenous bone regeneration at load‐bearing sites in a rat model of alveolar bone defects.

#### 6.2.3 Treatment for Osteochondral Injury

**FIGURE 13:**
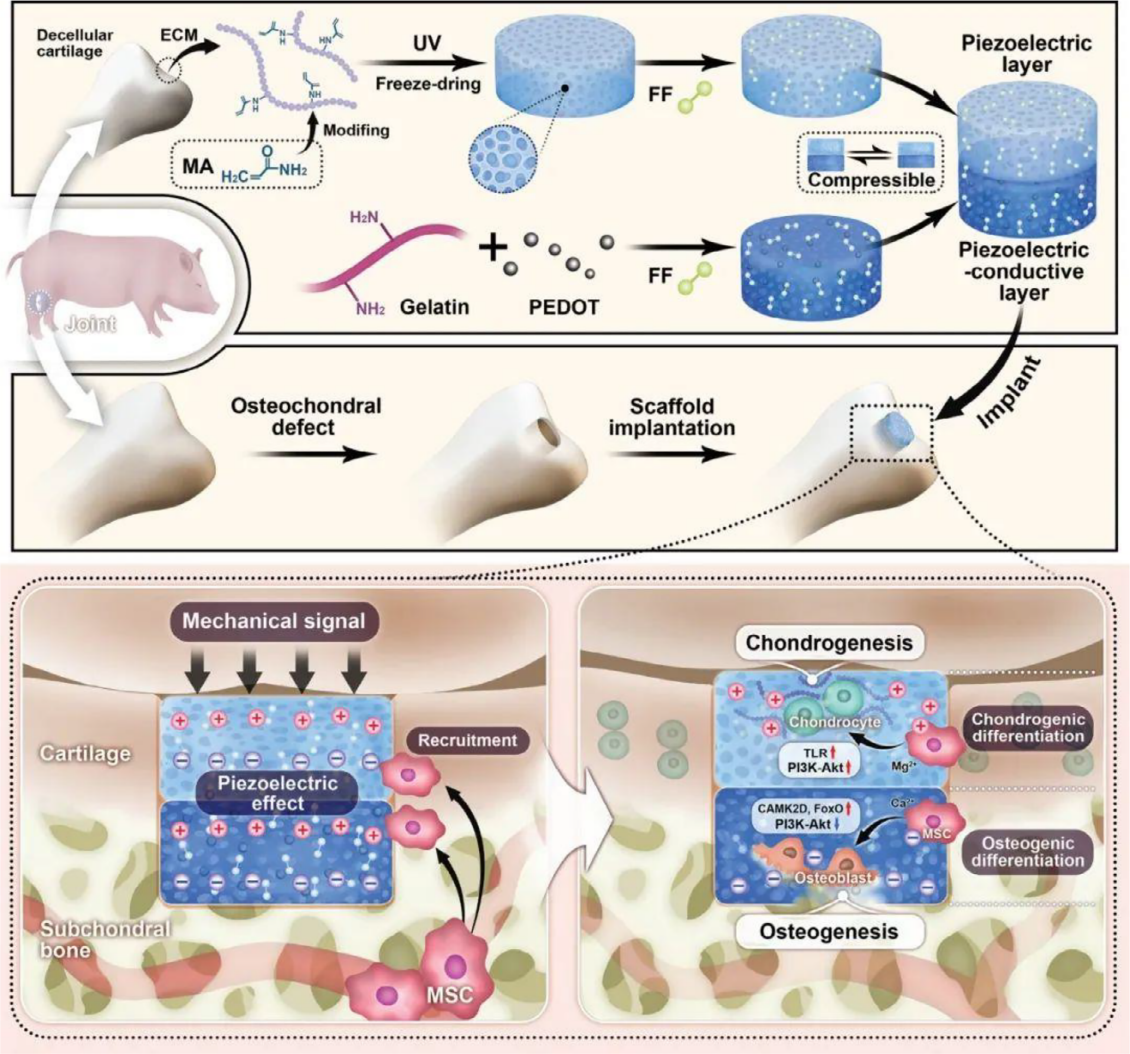
Illustration of biodegradable hydrogel scaffold with integrated piezoelectric and conductive properties for osteochondral repair. Reproduced with permission [171]. Copyright 2024, Wiley‐VCH GmbH.

Osteochondral injuries, prevalent in athletes and the elderly, present significant treatment challenges due to the limited regenerative capacity of cartilage, which lacks both blood vessels and nerves. Traditional treatments typically focus on symptom management using analgesics and anti‐inflammatory medications, which do little to repair the actual damage. While tissue engineering techniques like microfracture have been employed to aid osteochondral repair, they often struggle with effective delivery of bioactive factors, chemical modifications, and biomechanical optimization. Recently, electrical stimulation has been increasingly recognized for its potential to enhance bone and cartilage regeneration, given its pivotal role in various physiological processes. Research indicates that low‐voltage stimulation may aid cartilage differentiation and high voltage may favor osteogenesis. However, a major limitation of current bioelectrical stimulation materials is their lack of biodegradability. For example, materials containing heavy metals may possess favorable piezoelectric properties but are unsuitable for long‐term implantation due to toxicity concerns, and traditional piezoelectric polymers are not biodegradable, restricting their biomedical applicability. Addressing these challenges, Liu et al. developed a degradable piezoelectric‐conductive composite hydrogel scaffold, featuring a dual‐layer structure of piezoelectrically modified decellularized extracellular matrix and conductively modified gelatin [171]. This scaffold merges piezoelectric and conductive properties to promote bidirectional differentiation of cartilage and bone, creating a physiologically attuned osteochondral repair platform. In vitro results demonstrated the scaffold's excellent biocompatibility and its ability to significantly enhance cell migration, cartilage, and osteogenic differentiation (Figure 13). Further insights were provided by RNA sequencing, which identified crucial target molecules influenced by electrical stimulation. In vivo tests in large animal models revealed that the scaffold effectively met the high electrical output demands in bone areas and promoted stem cell migration and cartilage repair, particularly evidenced in a Parma pig model with osteochondral defects, showcasing its potential for clinical application. Electrical stimulation has emerged as a powerful tool to modulate membrane potential, activate intracellular signaling, and promote stem cell differentiation for tissue regeneration. To address challenges in spatially precise stimulation, Han et al. developed Piezo@CR microspheres that leverage US‐triggered piezoelectricity and stem cell‐targeting peptides for localized chondrogenic induction [172]. This system promotes calcium influx and activates p38 MAPK signaling, enabling precise electrical control of stem cell differentiation for cartilage repair.

#### 6.2.4 Nerve Regeneration

In addition to their roles in skin and bone tissue regeneration, piezoelectric biomaterials are also showing promise in the treatment of long‐gap peripheral nerve injuries. Neuronal cells inherently possess electrical activity, making the transmission of bioelectrical signals essential for nerve function recovery. Thus, an ideal peripheral nerve scaffold should not only exhibit a fine structure but also mimic the electrical characteristics of natural nerves. Piezoelectric biomaterials, which convert mechanical stress into electrical signals, are particularly suited for this purpose. They can activate various intracellular signaling pathways that are crucial for cellular activity and function, thereby supporting nerve tissue regeneration. This capacity to facilitate bioelectrical signal transmission makes piezoelectric materials promising candidates for enhancing the functional recovery of damaged nerves [189].

**FIGURE 14:**
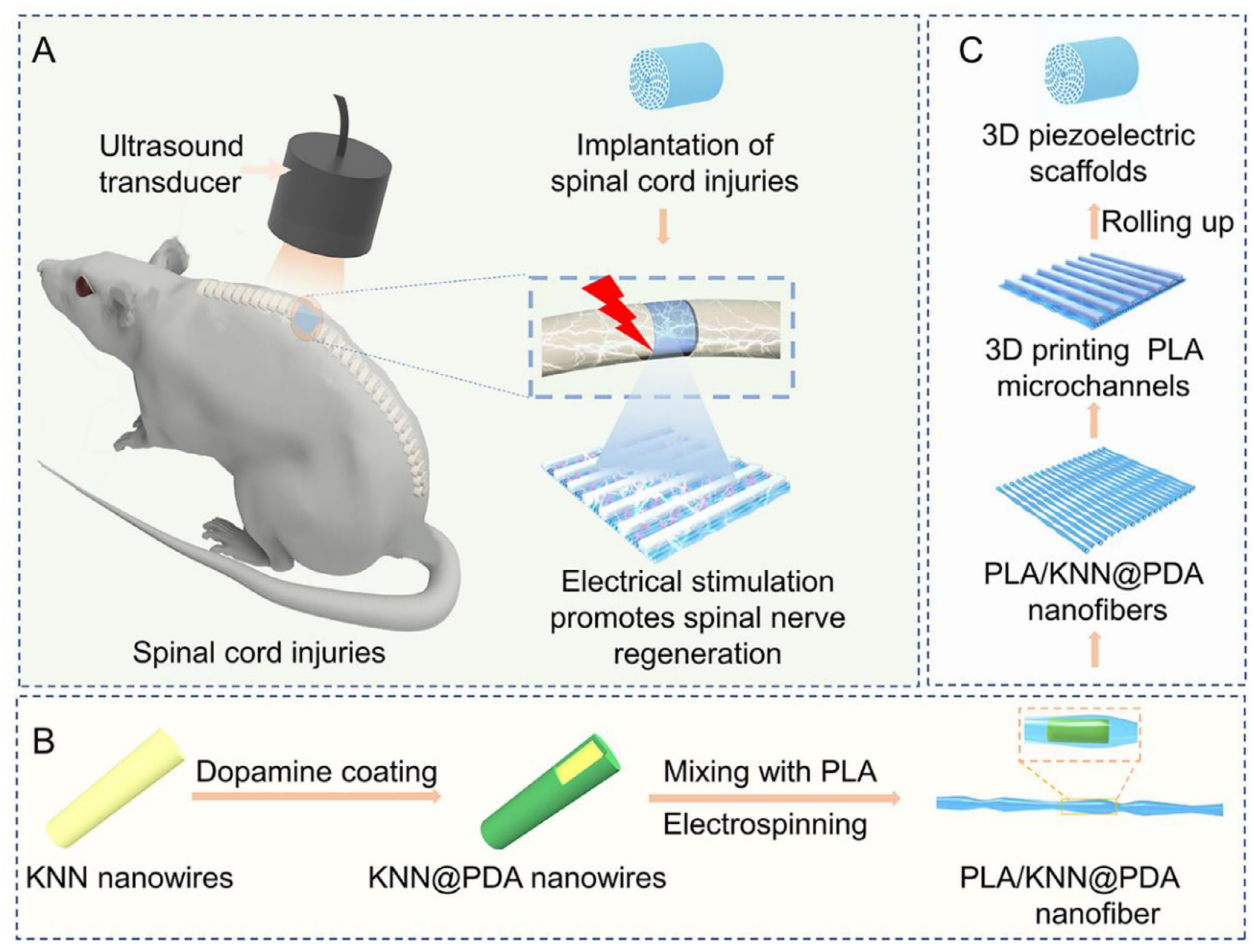
The diagram illustrates advancements in spinal cord injury treatment through wirelessly powered ES using biodegradable 3D piezoelectric scaffolds. (A) The US‐powered wireless ES from these innovative scaffolds significantly enhances neural regeneration in spinal cord injuries. (B) The production details of the PLA/KNN@PDA nanofibers used in the scaffolds. (C) An outline of the methods used to fabricate the 3D piezoelectric scaffolds from the PLA/KNN@PDA nanofibers. Reproduced with permission [174]. Copyright 2022, American Chemical Society.

In peripheral nerve regeneration, nerve conduits are vital for providing both mechanical support and electrical conductivity, aiding the self‐repair of damaged peripheral nerves. The bioelectrical conductivity of these conduits is crucial for maintaining nerve viability, promoting axon extension, and facilitating signal transmission. Leveraging the properties of piezoelectric biomaterials, which generate a piezoelectric potential under mechanical stress, innovative strategies have been employed to create an electrically conductive environment that accelerates nerve repair. For instance, Qian et al. designed a self‐powered nanogenerator scaffold made of a ZnO‐loaded polycaprolactone (ZnO/PCL) composite, which demonstrates significant proangiogenic and proneurogenic effects by mechano‐electrically stimulating Schwann cells under US stimulation [190]. This scaffold not only enhances motor recovery but also improves neural functions, particularly when combined with physical exercises like running, thereby fostering an optimal bioelectrical milieu for peripheral nerve regeneration. Chen et al. introduces a biodegradable, high‐performance 3D piezoelectric scaffold with US‐driven electrical stimulation capabilities for spinal cord injury repair [174]. Constructed from electrospun PLLA nanofibers embedded with potassium sodium niobate nanowires, the scaffold enables programmable, on‐demand electrical stimulation in vivo. Demonstrated in a rat model, this innovative scaffold enhances motor function recovery and promotes neural stem cell differentiation and angiogenesis at the injury site (Figure 14). Similarly, Shan et al. loaded MSCs onto a membrane composed of piezoelectric PLLA fibers, and mimicked the extracellular matrix by adding hydrogels and neuroepithelial cells [175]. Under U‐induced excitation, the electric field formed by the piezoelectric potential on the surface of PLLA membrane mimics the endogenous electrical environment during embryonic development; In addition, electrical stimulation promotes the differentiation of mesenchymal stem cells, enhances their paracrine effect, and then induces the recruitment and differentiation of endogenous stem cells, ultimately promoting axonal growth.

Where gaps exceed 8 mm and direct neurorrhaphy is not viable, and autologous nerve transplantation remains the standard of care [191]. However, the use of autologous grafts is constrained by limitations such as the scarcity of donor nerves, donor site morbidity, and potential surgical complications [192]. Although various biophysical strategies have been explored for treating peripheral nerve defects, none have yet matched the efficacy of standard autografts. Addressing this challenge, Pi et al. introduced a sono‐electro‐mechanical therapy that leverages electrospinning technology to create linearly aligned piezoelectric fibers from polycaprolactone and PVDF. This innovative approach significantly enhanced the regenerative functions of Schwann cells and neuronal cells in vitro. Their method proved effective in a rat model with a 15 mm sciatic nerve gap, restoring motor functions, muscle histology, and axonal maturity to levels comparable to autograft treatments [173].

Numerous nervous system diseases (NSD), including nerve trauma and neurodegenerative disorders, significantly contribute to global disability and mortality rates [193]. Research has highlighted the benefits of electrical stimulation in treating these conditions, particularly through its ability to enhance the levels of brain‐derived neurotrophic factor (BDNF) and its receptor, tropomyosin receptor kinase B (TrkB). This interaction triggers a calcium‐dependent mechanism that leads to the upregulation of regeneration‐associated genes through cyclic adenosine monophosphate (cAMP) pathways. Such cellular and molecular activations are crucial for promoting axonal repair and preventing the collapse of growth cones, crucial steps in nerve regeneration. In this context, piezoelectric biomaterials show promising potential as they can generate necessary electrical stimulation in response to mechanical stress applied to damaged nerves [194]. This innovative approach harnesses natural bodily movements to aid in nerve recovery, offering a non‐invasive and dynamic method to enhance neural healing processes. Stem cell‐based therapies are increasingly recognized as a promising approach for treating NSD. Liu et al. developed an innovative micromotor by incorporating biodegradable Spirulina platensis with Fe_3_O_4_ and BTO nanoparticles through electrostatic adsorption, enabling both magnetic maneuverability and piezoelectric functionality [195]. This micromotor, controlled by a low‐intensity rotating magnetic field, can selectively target neural stem cells, with the piezoelectric properties of BTO nanoparticles converting US energy into electrical stimulation. This stimulation activates intracellular calcium channels and signaling pathways that encourage neural stem cell differentiation.

Traumatic brain injury (TBI) often results in irreversible neurological damage due to the limited regenerative capacity of neurons. Neural stem cells (NSCs) offer therapeutic potential but are hindered by low efficiency in differentiation and proliferation. Recent studies have demonstrated that piezoelectric stimulation can enhance NSC activity. Notably, Wang et al. developed barium titanate–reduced graphene oxide (BTO/rGO) hybrid piezoelectric nanostickers that adhere to NSC membranes and, upon ultrasound stimulation, generate localized electric fields to promote neuronal differentiation [196]. This process involves activation of voltage‐gated calcium channels and the downstream Ca^2+^/CaMKII/CREB signaling cascade. In a TBI rat model, transplantation of NSCs functionalized with BTO/rGO nanostickers significantly improved tissue regeneration and neurological function, highlighting the promise of piezoelectric nanotechnology in neural repair strategies.

In summary, the application of piezoelectric biomaterials in neural regeneration has emerged as a rapidly evolving and promising field. By converting mechanical stimuli into localized electric signals, piezoelectric platforms offer a bioinspired strategy to modulate neuronal behavior, activate mechanosensitive ion channels, and guide neural stem cell differentiation and maturation. Advances in biomaterial design have significantly improved the biocompatibility, responsiveness, and integration of these materials within neural tissues. Moreover, the combination of piezoelectric stimulation with stem cell therapy, targeted drug delivery, and non‐invasive modalities such as ultrasound has opened new avenues for the treatment of complex neurological injury. In future, the integration of piezoelectric materials with intelligent bioelectronics and precision neuromodulation technologies is expected to further accelerate breakthroughs in neuroregenerative medicine.

## 7 Conclusion and Outlook

As a recently emerging class of functional biomedical materials, piezoelectric platforms have garnered considerable interest due to their low cost, ease of preparation, and stable performance. As a special branch of nanocatalytic medicine [197], piezocatalytic therapy relies on mechanically triggered advanced oxidation processes to generate ROS through interactions between piezoelectric nanomaterials and molecules such as oxygen and water. This review summarized the classification, modification strategies, and biomedical applications of piezoelectric biomaterials, including their roles in wound healing, osteomyelitis treatment, tissue regeneration, and oral infection management. In the future, piezoelectric biomaterials are expected to be increasingly integrated into biosensing and medical monitoring platforms. Notably, flexible and biodegradable piezoelectric materials hold promise for incorporation into wearable electronics for real‐time health monitoring and personalized healthcare feedback [198]. Despite these exciting developments, piezoelectric biomedical applications remain in their infancy and face several critical challenges:.

1. **Safety evaluation**: For successful clinical translation, it is essential to assess the biological safety and long‐term biocompatibility of piezoelectric biomaterials. Key concerns include cytotoxicity, in vivo distribution and metabolism, and the retention of materials in specific microenvironments [199]. Copolymers have emerged as safer alternatives to toxic lead‐based ceramics. However, prolonged implantation may still lead to metabolic disturbances or chronic inflammation. The development of biodegradable piezoelectric materials is therefore a promising strategy. While electrical stimulation has shown benefits for promoting tissue regeneration and cell differentiation [6], inappropriate stimulation may negatively impact cell viability and proliferation [200]. Although some bio‐piezoelectric platforms have demonstrated stimulatory effects on cellular behavior, the safe electrical signal range remains undefined. Systematic evaluations are required to determine safety thresholds for various tissues and cell types.
2. **Material design**: Optimizing piezoelectric efficiency is central to advancing clinical applications of these materials. Strategies include refining synthesis conditions, introducing crystal defects, and employing self‐assembly techniques to optimize morphology and electromechanical performance. Additionally, artificial intelligence and machine learning are increasingly used to predict high‐performance piezocatalytic materials, elucidate catalytic mechanisms, and streamline materials development [201].
3. **Synergy with other therapies**: While most studies focus on the physical properties of piezoelectric materials, their chemical interactions and potential synergy with other therapeutic modalities are underexplored. Combining piezoelectricity with other functionalities, such as immunomodulation or redox catalysis, may lead to novel multimodal treatment platforms. For instance, piezoelectric materials capable of generating ROS in the tumor microenvironment can be coupled with immunotherapies to enhance anticancer responses through a synergistic mechanism [202].
4. **Mechanism elucidation**: Despite numerous studies demonstrating the biomedical effects of piezoelectric materials, the underlying mechanisms—particularly in vivo—remain poorly defined. Most explanations attribute therapeutic outcomes to catalytic or electrical effects, often overlooking the direct interactions between nanomaterials and biological systems. Advanced imaging, molecular labeling, and real‐time in vivo analysis are essential for uncovering these mechanisms.
5. **US power concerns**: US is frequently used to activate piezoelectric materials; however, excessive US energy may cause tissue damage due to cavitation effects. Future research should focus on lowering the required US intensity by enhancing the intrinsic piezoelectric properties of materials or exploring alternative mechanical stimulation strategies.
6. **Process standardization**: Clinical translation requires scalable, reproducible, and precisely controlled fabrication of piezoelectric platforms. A major barrier is the lack of standardized evaluation protocols, especially given the variability introduced by external mechanical stimuli. Parameters such as reactor vessel size, shape, and material can significantly affect US intensity and catalytic output.
7. **Implantable device challenges**: Organic piezoelectric materials are attractive for implantable devices due to their flexibility and biocompatibility [203], but their relatively low piezoelectric coefficients limit performance. Strategies to control polarization orientation and improve long‐term material stability, such as surface coatings and advanced synthesis techniques, are critical. Additionally, future materials must conform to complex tissue geometries while maintaining mechanical integrity [204].
8. **Clinical translation**: Although many experimental results are encouraging, long‐term biocompatibility remains to be validated. Risks such as solvent residues in PVDF and Ba^2+^ leaching from BTO must be addressed. Physiological challenges, including corrosion by body fluids and degradation under dynamic mechanical stress, may compromise long‐term functionality. Furthermore, maintaining consistent crystal orientation during large‐scale production is difficult. Future work should include surface modification to mitigate foreign body responses and accelerated aging studies to simulate long‐term in vivo conditions.

In conclusion, the piezoelectric nanoplatforms based on piezoelectric catalytic medicine have both great potential and great challenges to be explored and solved. Many new piezoelectric biomaterials and systems will be developed in the future as a result of intensive multidisciplinary research. These materials and systems are anticipated to be refined and progressively used in clinical settings, thereby advancing medical innovation and improving human health.

## Author Contributions

Yongbin Mou, Dongliang Yang and Heng Dong developed the concept. Ao He, Meng Ding, Yu Zhang, Zhuo Dai, Qiang Li, Weijun Xiu and Yanling Hu participated in the literature analysis and the processes of Figures andvisualizations. The work was supervised by Dongliang Yang and Heng Dong. Wenxuan Mao and Xiaoye Li drafted the manuscript. Heng Dong and Dongliang Yang revised the manuscript.

## Conflicts of Interest

The authors declare no conflicts of interest.
